# Supplementary figures and images for: The assembly of neutrophil inflammasomes during COVID-19 is mediated by type I interferons
Source: PLoS Pathog. 2024 Aug 22;20(8):e1012368. doi: 10.1371/journal.ppat.1012368 (PMC11340896; doi:10.1371/journal.ppat.1012368)

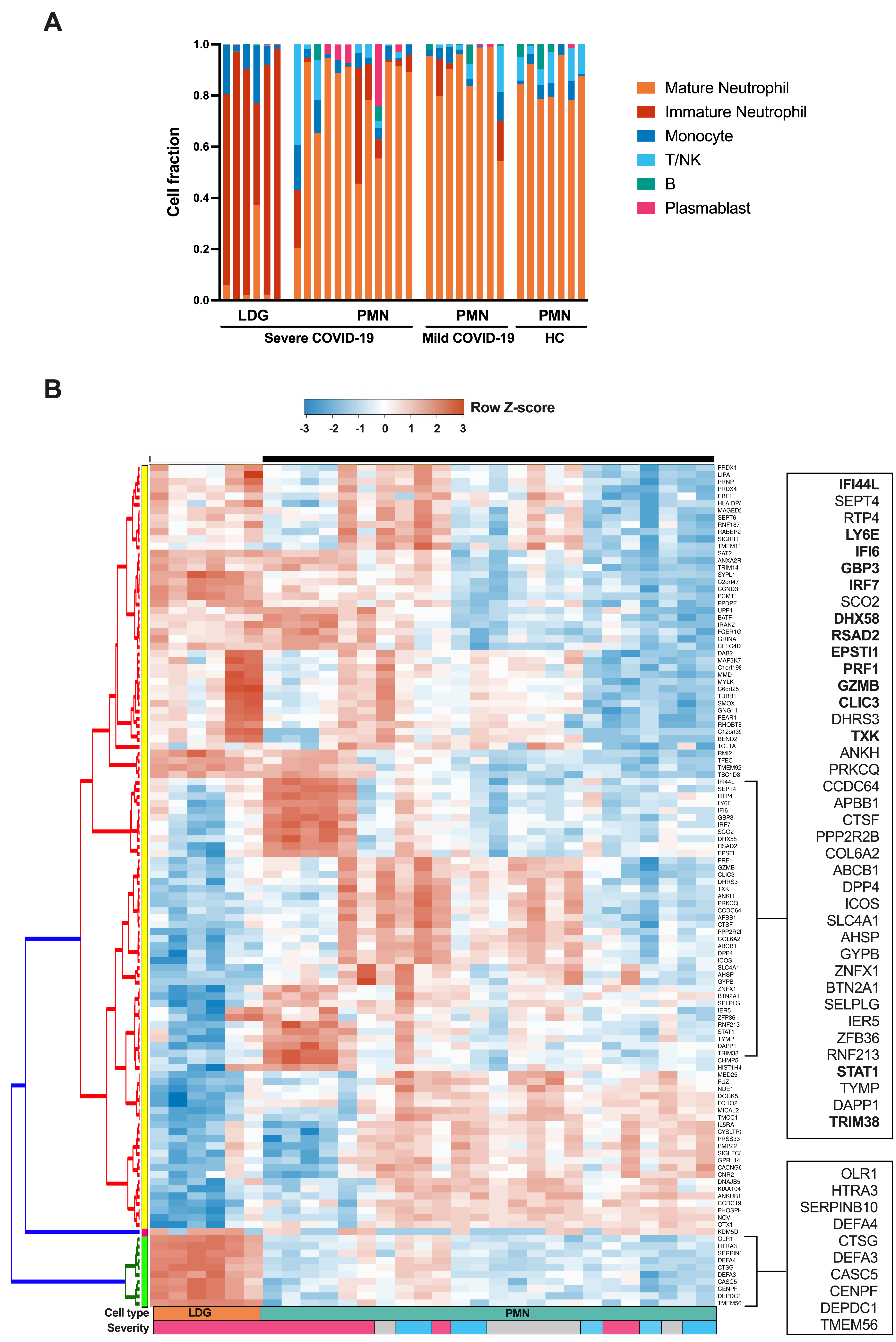

Supplement: S1 Fig — (A) Deconvoluted RNA-seq data. The cellular composition in isolated PMN and LDG fractions was estimated using CIBERSORTx through the identification of cell populations based on RNA-seq. The bar plots in the figure represent the cell composition of each RNA-seq sample, offering insights on sample purity. (B) Heatmap of the top 118 differentially expressed genes between PMNs from healthy controls, mild and severe COVID-19, as well as LDGs from severe disease, identified by unsupervised ICGS analysis based on correlation, using AltAnalyze software. IFN-related genes, identified by GENESHOT, are shown in bold. (TIFF) [file ppat.1012368.s001.tiff]

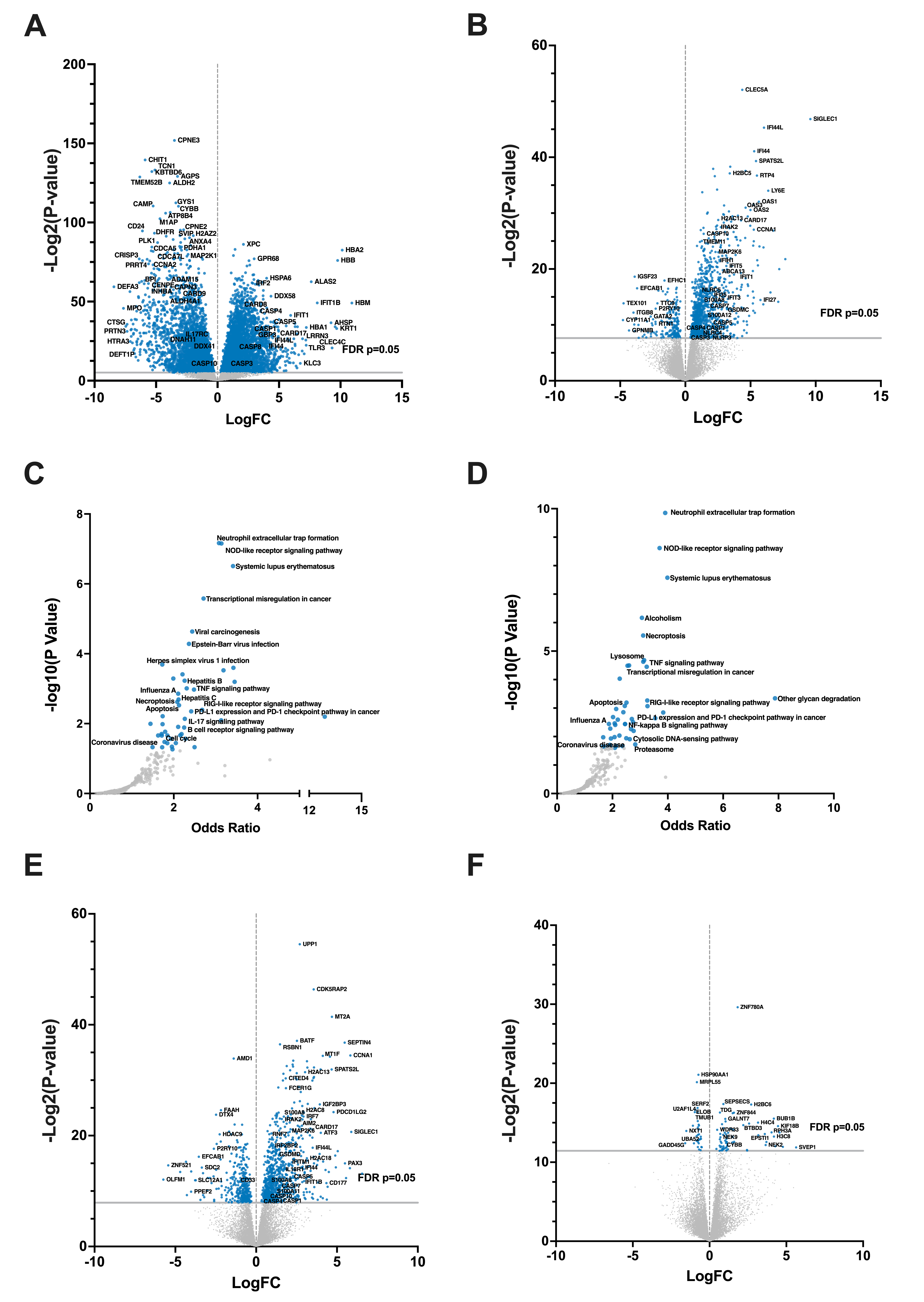

Supplement: S2 Fig — (A-B) Volcano plots of DEGs between severe COVID-19 PMNs versus (A) severe COVID-19 LDGs and (B) HC PMNs. (C-D) Volcano plots of enriched gene sets in severe COVID-19 PMNs versus (C) HC PMNs and (D) severe COVID-19 LDGs, using KEGG database. Each point represents a single gene set, where the x-axis measures its odds ratio, while the y-axis shows its -log10(p-value). (E-F) Volcano plots of (E) severe COVID-19 PMNs versus mild COVID-19 PMNs and (F) mild COVID-19 PMNs vs HC PMN. For all panels, blue points represent significant terms (adjusted p-value < 0.05), while smaller gray points represent non-significant terms. DEG = differentially expressed genes. (TIFF) [file ppat.1012368.s002.tiff]

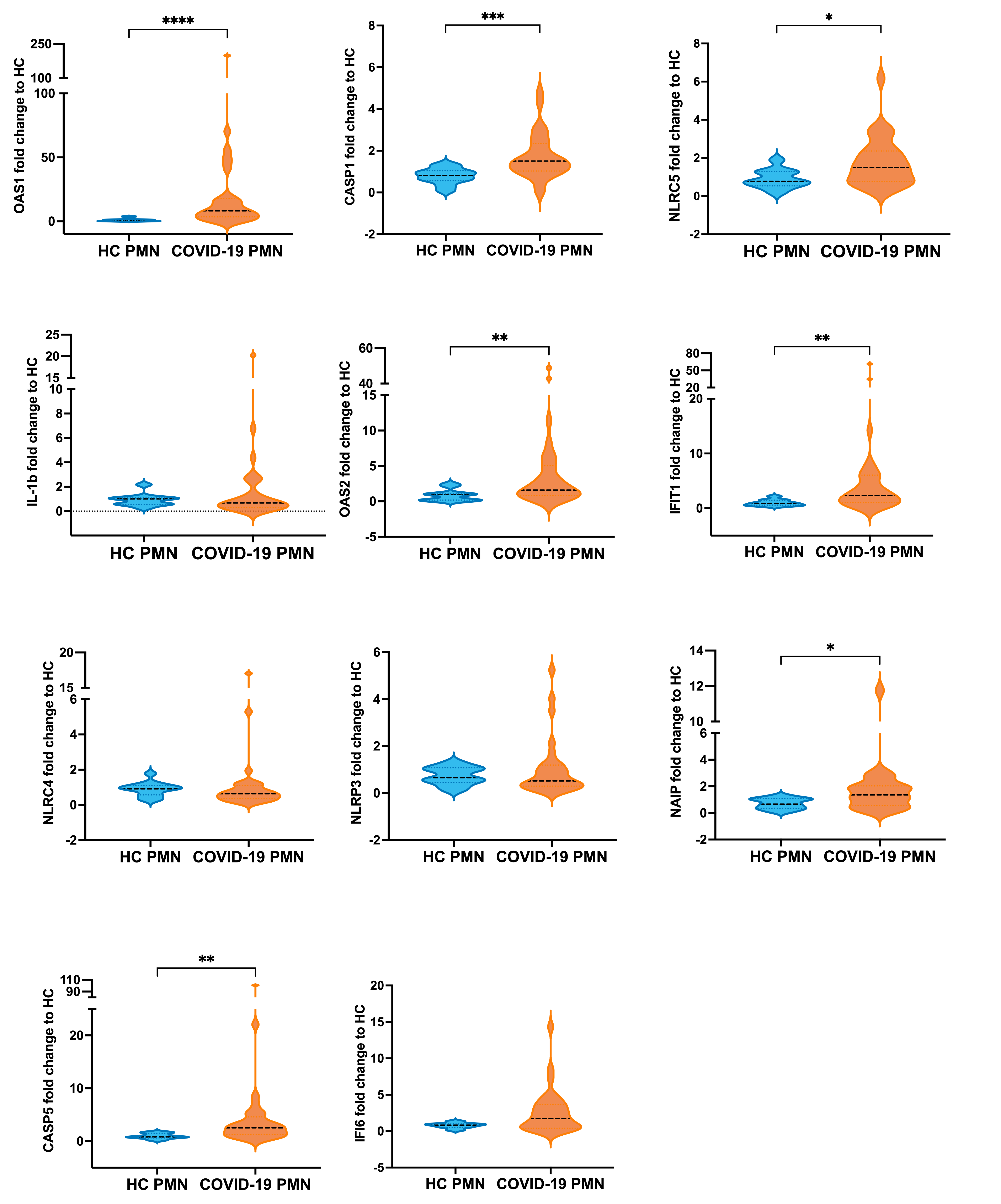

Supplement: S3 Fig — RNA was extracted from isolated HC PMNs (n = 8–13) versus severe COVID-19 PMNs (n = 29–32) and subjected to comparative RT-qPCR using specific primers for OAS1, OAS2, IFIT1, IFI16, caspase1, caspase5, IL1B, NLRC4, NLRC5, NLRP3 and NAIP. *p < 0.05, **p < 0.01, ***p < 0.001 and **** p < 0.0001. P values calculated with Mann-Whitney U-test. Data presented as mean ± SD. (TIFF) [file ppat.1012368.s003.tiff]

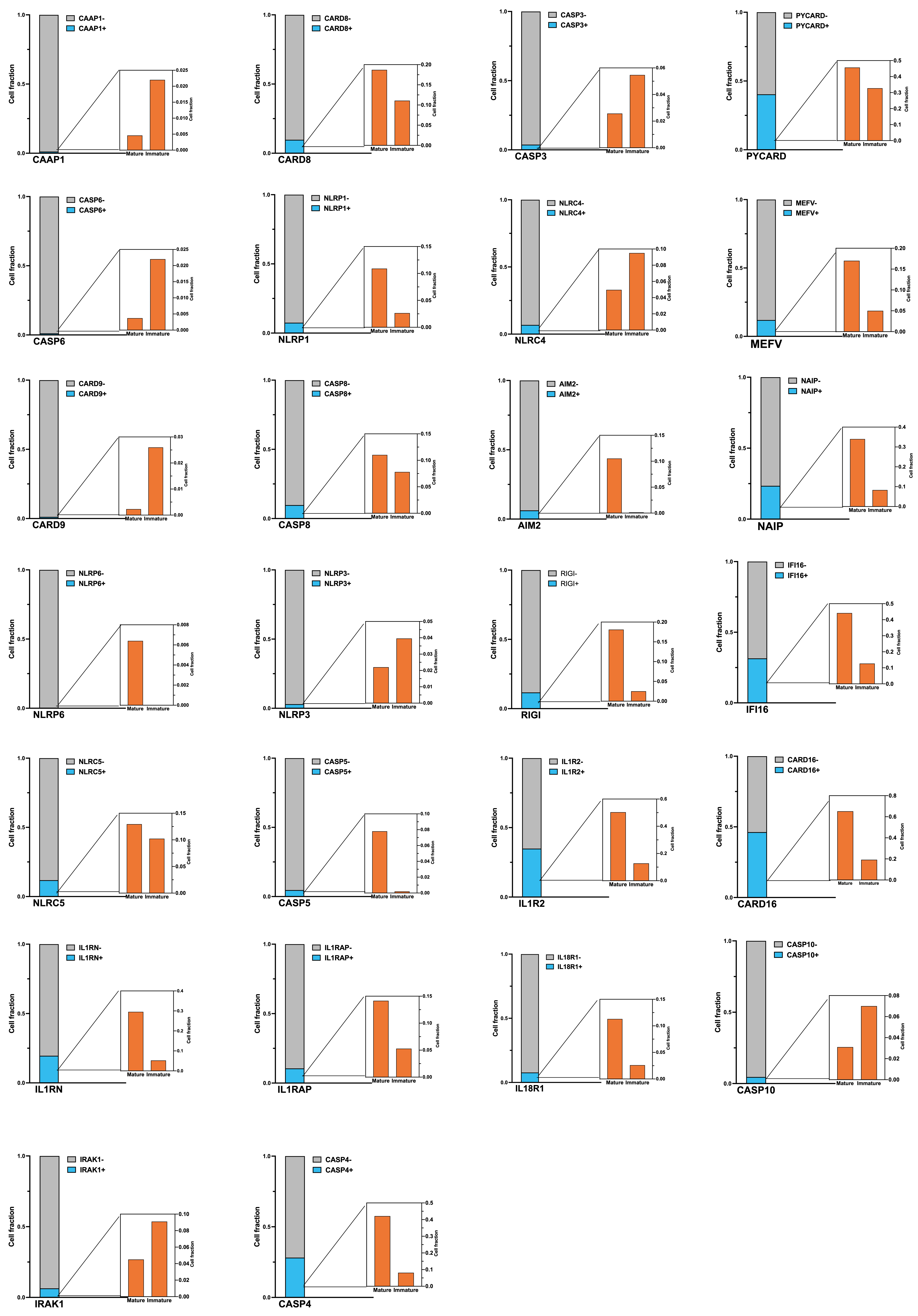

Supplement: S4 Fig — The fraction of mature and immature neutrophils cells expressing 17 inflammasome related genes identified in Fig 2D (shown in black and blue, respectively) are shown in a bar graph. For each gene, the proportion of expressing cells is shown in light blue, while the proportion of negative or not-expressing cells is shown in gray. Zoomed-in bar graph depicts the proportion of mature and immature cells expressing each gene. (TIFF) [file ppat.1012368.s004.tiff]

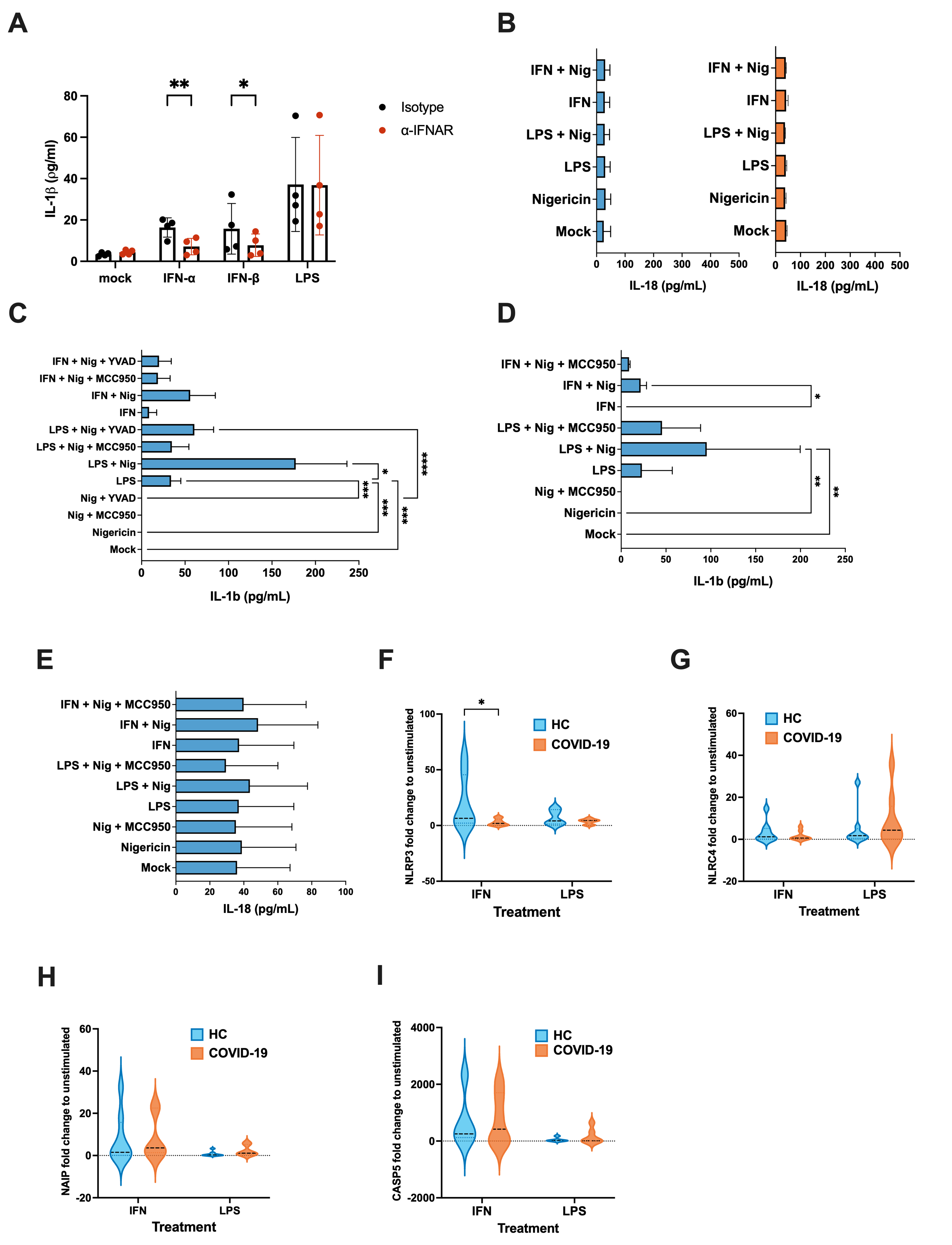

Supplement: S5 Fig — (A) HC PMNs (1 million/ml) were primed for 4 hr by low dose IFN-α, low dose IFN-β (both 2.7*103 IU/ml) or 20 ng/ml LPS followed by 2.5 μM nigericin activation for 4 hr. IL-1β release was measured by ELISA and the assays were performed in the presence of either α-IFNAR1 or mouse IgG as control (both 100 μg/ml) (n = 5). *p < 0.05 and **p < 0.01. P values calculated using two-way ANOVA with Šídák multiple comparison test. (B) IL-18 (n = 2–3 HC PMN and 3 COVID-19 PMN) was measured from supernatants by ELISA following LPS or IFN-I priming (4 h) and subsequent nigericin activation (4 h). (C-E) Effect of different inflammasome specific inhibitors in cytokine secretion. (C) Effect of inflammasome inhibitor MCC950 (2 μg/ml) and YVAD (20 μg/ml) on LPS or IFN-I primed (4 h) and nigericin activated (4 h) IL-1β secretion in the supernatant of healthy control PMNs (n = 8). (D-E) Effect of inflammasome inhibitor MCC950 (2 μg/ml, added simultaneously with nigericin) on LPS or IFN-I primed (4 h) and nigericin activated (20 h). *p < 0.05, **p < 0.01, ***p < 0.001, **** p < 0.0001. P values calculated with Kruskal-Wallis test. Data presented as mean ± SD. IFN = interferon type I, LPS = lipopolysaccharide, Nig = nigericin, YVAD = tetrapeptide caspase1 inhibitor Tyr-Val-Ala-Asp. (F-I) Gene expressions in HC and COVID-19 PMNs after LPS or IFN-I stimulation. A comparison of gene expression in isolated healthy control PMNs versus COVID-19 PMNs after ex vivo stimulation with LPS or IFN-I. Extracted RNA was subjected to comparative RT-qPCR using specific primers for NLRP3, NLRC4, NAIP and CASP5 (n = 4–8 for HC PMN and 6–9 for COVID-19 PMN). *p < 0.05. Two-way ANOVA with Tukey’s multiple comparison test was applied. Data were presented as mean ± SD. (TIFF) [file ppat.1012368.s005.tiff]

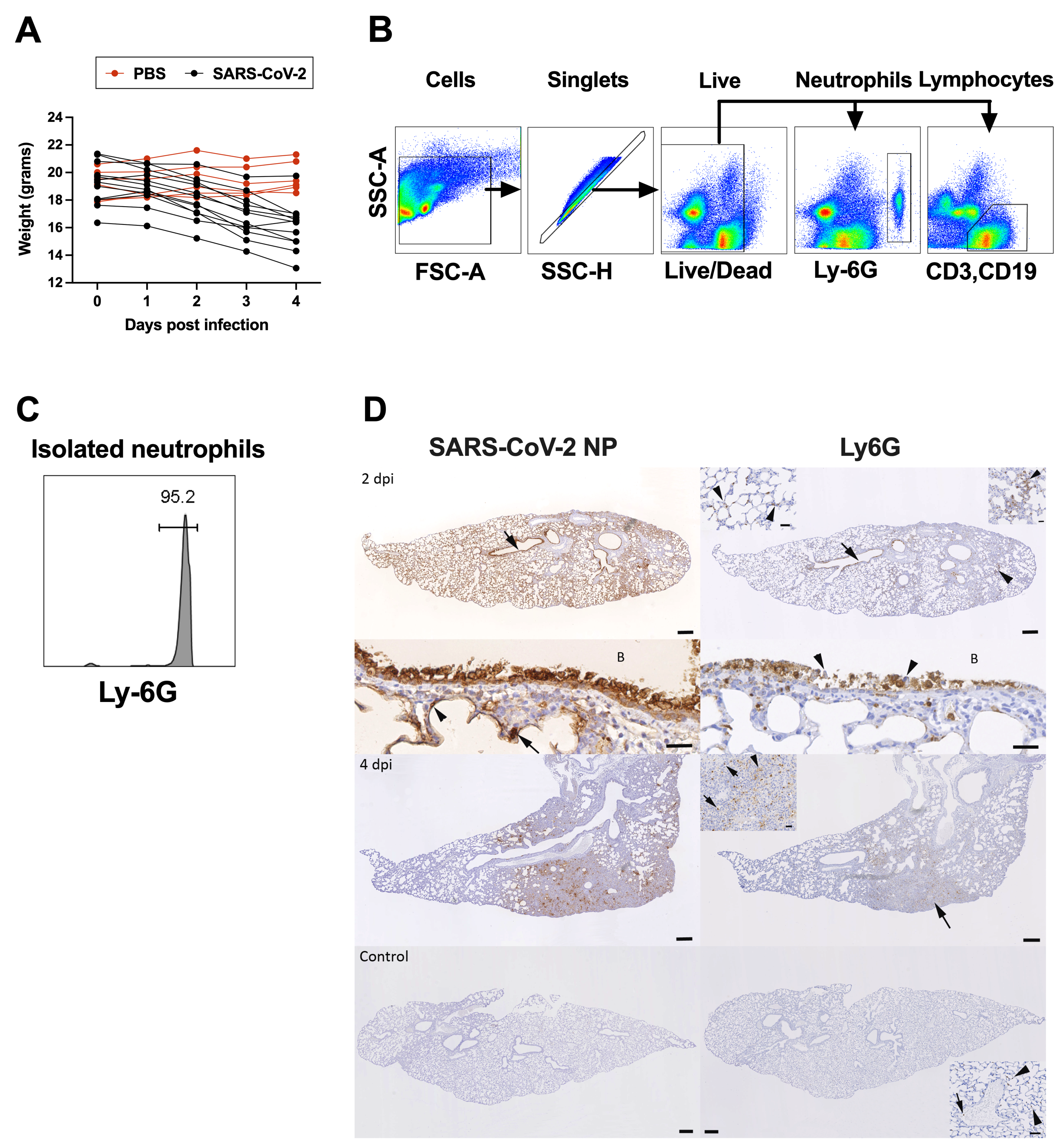

Supplement: S6 Fig — Female BALB/c mice were intranasally inoculated with 5*105 TCID50 SARS-CoV-2 MaVie strain or PBS as control and euthanized at 2 dpi or 4 dpi. (A) Daily tracking of animal weight performed throughout the experiment (n = 12 for SARS-CoV-2 infected animals, n = 6 for PBS-inoculated animals). The weights of the mice euthanized at 2 dpi (n = 26) did not show significant differences and are not reported. (B) Gating strategy to analyze Ly6G+ neutrophils in mouse lung single cell suspensions. Side scatter area (SSC-A) versus forward scatter area (FSC-A) plot followed by side scatter area versus height (SSC-A vs SSC-H) plot were used for the identification of single cells. BV605 yellow live/dead dye was used to discriminate dead cells, from which CD3/CD19+ lymphocytes and Ly6G+ neutrophils were gated as shown. (C) Representative histogram showing the percentage of Ly6G+ cells after isolation from lung single cell suspension using Ly6G-binding magnetic beads. (D) Left column: immunohistochemistry for SARS-CoV-2 nucleoprotein; right column: immunohistochemistry for Ly6G (neutrophil marker), hematoxylin counterstain. Bars = 500 μm (large images) and 50 μm (insets). At 2 dpi (top), the arrow points at a bronchus with viral antigen expression in epithelial cells. A close–up of the bronchus (bottom; B: bronchial lumen) shows degenerated and slough off antigen positive epithelial cells. Adjacent alveoli exhibit viral antigen expression in typeI (arrowhead) and typeII (arrow) pneumocytes. The overview (top) shows neutrophils between the infected bronchial (arrow) epithelial cells, in parenchymal areas (arrowhead; right inset) and in capillaries (arrowheads). A close-up of the bronchus (bottom; B: bronchial lumen) highlights numerous neutrophils between degenerate (arrowheads) epithelial cells. At 4 dpi (middle), there are focal areas with antigen expression in alveolar epithelial cells and infiltrating macrophages. Neutrophils are present among the infiltrating cells (arrow) as indi [file ppat.1012368.s006.tiff]

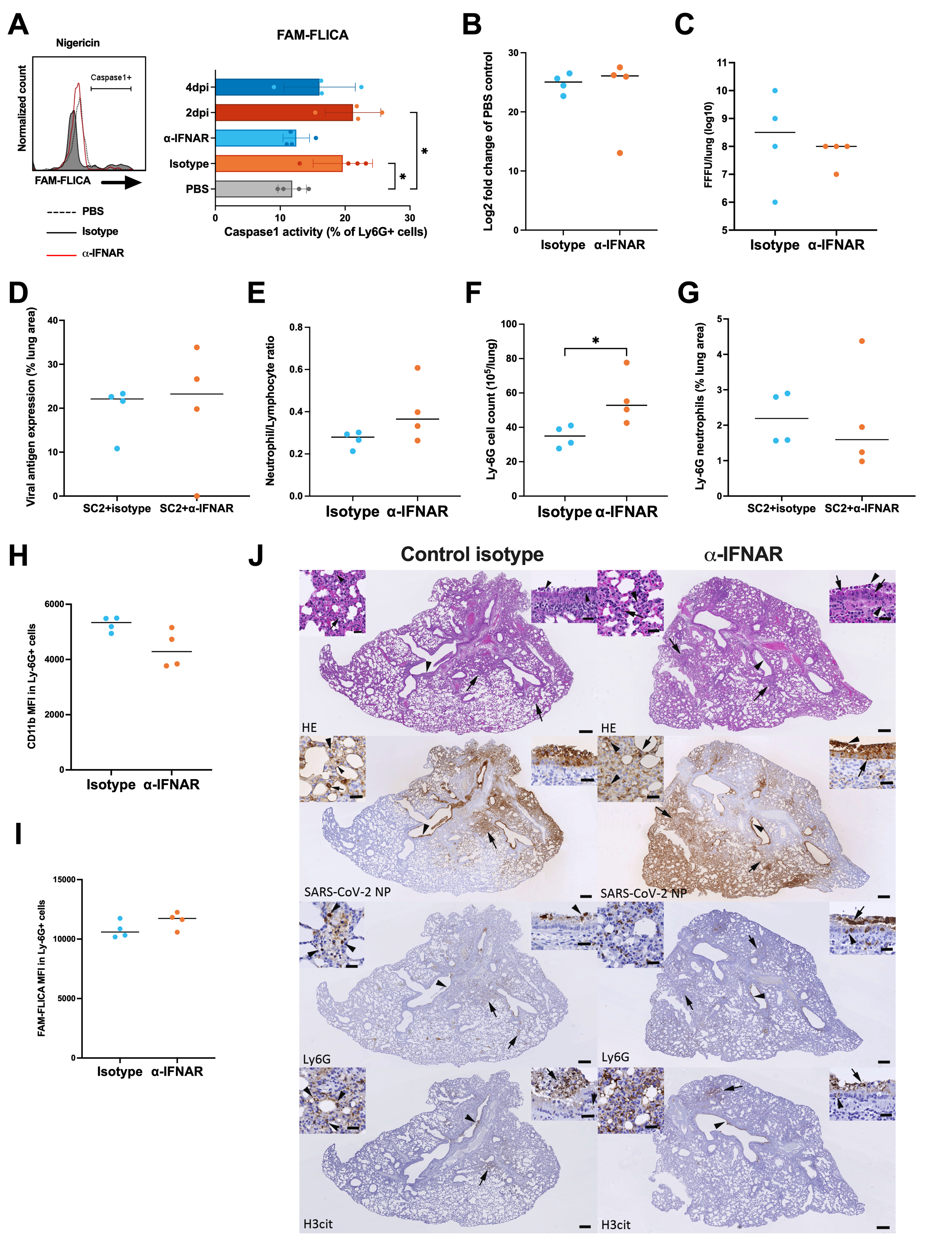

Supplement: S7 Fig — Female BALB/c mice were intranasally inoculated with 5* 105 TCID50 SARS-CoV-2 MaVie strain or PBS as control. Mice were intraperitoneally inoculated with 250 μg anti-IFNAR or IgG1 isotype control directly after infection with SARS-CoV-2 and lung neutrophils isolated at 2 dpi (including also intranasally PBS-inoculated control mice without intraperitoneal injection) (A) Quantification of caspase1 positive cells in nigericin-activated isolated Ly6G neutrophils stained by FAM-FLICA. Representative histogram is shown. * p < 0.05. P values calculated using one-way ANOVA with multiple comparison test (Holm-Šídák correction). (B) RNA was isolated from mouse lungs and subjected to RT-qPCR targeting the replication-intermediate subgenomic E gene and GAPDH as housekeeping gene. RNA levels were assessed based on cycle threshold Ct levels. The expression levels of the target gene SubE were measured and normalized to GAPDH levels using the comparative Ct method (ΔΔCt). The fold change values were calculated by the formula 2^(-ΔΔCt), representing the relative gene expression compared to the PBS mock-infected control (in which subE was undetectable but set to 40 Ct). No significant differences are seen between the two groups, assessed with Welch’s t-test. (C) Infectious virus was calculated from supernatants of lung single cell suspensions of infected mice as fluorescence focus forming units (FFU) in Vero E6 cells. (D) Quantification based on morphometric analysis that determines the area of immunolabelling for SARS-CoV-2 nucleoprotein in relation to total tissue area. (E) Quantification of Ly6G neutrophil/lymphocyte ratio in lung single cell suspensions by flow cytometry. (F) Quantification of Ly6G cell counts extrapolated per lung in single cell suspensions by flow cytometry (G) Quantification of Ly-6G based on morphometric analysis that determines the area of immunolabelling for Ly6G in relation to total tissue area in mock-infected controls. (H) Quantification of median fluore [file ppat.1012368.s007.tiff]

**supernatant**

**cells**

**anti-actin**

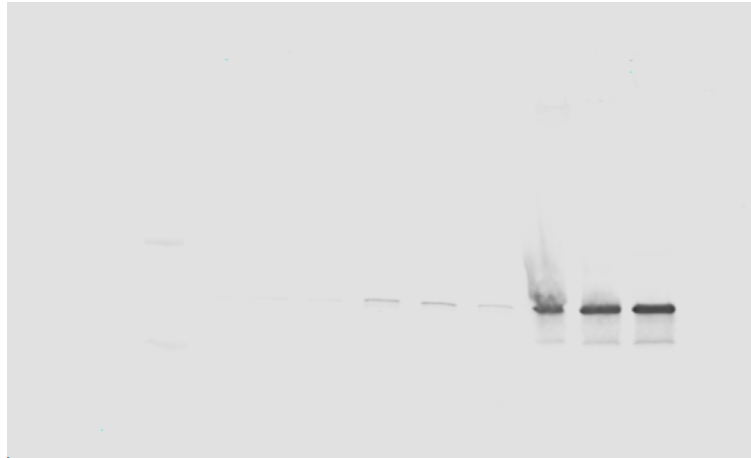

**anti-actin  
+ anti-IL1 $\beta$**

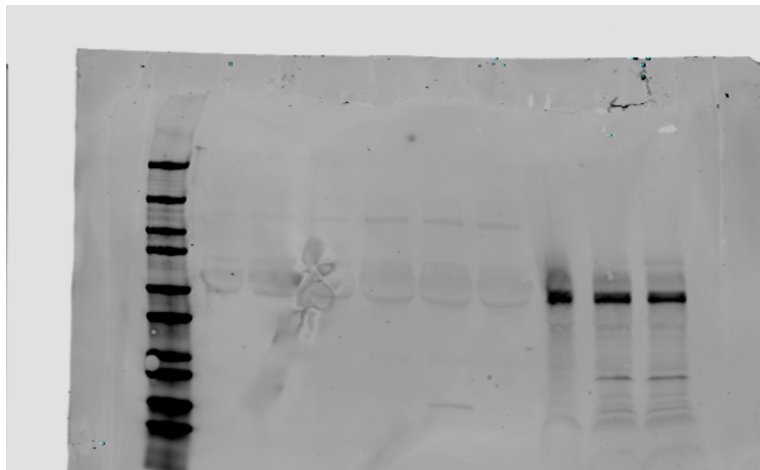

marker - LPS IFN - LPS IFN - LPS IFN

**nigericin**

Supplement: S1 Source Data — Western blots from supernatants and cell lysates were performed first for actin and then for IL-1β on the same membrane as indicated. Information for Fig 5A. (PDF) [file ppat.1012368.s011.pdf]
