# Supplementary material for: The assembly of neutrophil inflammasomes during COVID-19 is mediated by type I interferons
Source: PLoS Pathog. 2024 Aug 22;20(8):e1012368. doi: 10.1371/journal.ppat.1012368 (PMC11340896; doi:10.1371/journal.ppat.1012368)
Supplement: S2 Table — (DOCX) [file ppat.1012368.s009.docx]

**Supplementary Table S2. qPCR primer sequences: gene-specific forward and reverse primers.**

| Gene | Forward primer | Reverse primer |
| --- | --- | --- |
| AIM2 | GGCCCAGCAGGAA TCTATCAG | GAAGGGCTTCTTTGCTTTCAGTAC |
| NAIP | AAGGGATTTGTTGACATAACGGG | CAGCCGTAGTTCTTCGTAAGC |
| NLRC5 | ACAGCATCCTTAGACACTCCG | CCTTCCCCAAAAGCACGGT |
| IL1B | CACATGGGATAACGAGGCTT | TCCAGCTGTAGAGTGGGCTT |
| CASP1 | GCTTTCTGCTCTTCCACACC | TCCTCCACATCACAGGAACA |
| CASP4 | CAAGAGAAGCAACGTATGGCA | AGGCAGATGGTCAAACTCTGTA |
| CASP5 | TTCAACACCACATAACGTGTCC | GTCAAGGTTGCTCGTTCTATGG |
| IFI16 | TCCTCAGATGCCTCCATCAAC | CAGGTTCAGTCTTCAGTCTTGGT |
| IFIT5 | TAAAAAAGGCCTTGGAGGTG | CCAGGTCTGTGTAGGCAAAT |
| IRF7 | GCTGGACGTGACCATCATGTA | GGGCCGTATAGGAACGTGC |
| IFIT1 | GCGCTGGGTATGCGATCTC | CAGCCTGCCTTAGGGGAAG |
| IFI6 | GGTCTGCGATCCTGAATGGG | TCACTATCGAGATACTTGTGGGT |
| OAS1 | TGTCCAAGGTGGTAAAGGGTG | CCGGCGATTTAACTGATCCTG |
| OAS2 | CTCAGAAGCTGGGTTGGTTTAT | ACCATCTCGTCGATCAGTGTC |
| GAPDH | TTGGCTACAGCAACAGGGTG | GGGGAGATTCAGTGTGGTGG |
