# Supplementary material for: The assembly of neutrophil inflammasomes during COVID-19 is mediated by type I interferons
Source: PLoS Pathog. 2024 Aug 22;20(8):e1012368. doi: 10.1371/journal.ppat.1012368 (PMC11340896; doi:10.1371/journal.ppat.1012368)
Supplement: S3 Table — (DOCX) [file ppat.1012368.s010.docx]

**Supplementary Table S3**. **Histological changes as well as SARS-CoV-2 nucleoprotein and RNA expression in female BALB/C mice infected with SARS-CoV-2.**

**A.** **Experiment 1**. Infected animals were euthanized and examined at 2 and 4 days post infection.

| **Infection, dpi**  [Animal no] | **Histological changes, viral antigen and Ly6G expression^1^ in lungs** | **Viral subE (and GAPDH) RNA Cts^2^** | **Viral antigen expression (% lung area)^3^** | **Ly-6G neutrophils (% lung area)^3^** |
| --- | --- | --- | --- | --- |
| **5 x** **10^5 TCID50, 2 dpi**  [1.1.1] | **HE:** bronchioles with abundant degen EC (in place and sloughed off); parenchymal areas with type II pn activation, occ degen AEC and increased cellularity  **vAg:** extensive expression in bronchus/bronchioles (almost all BEC, some also degen); most alveoli with pos AEC  **Ly6G:** increase in NL in lumen of vessels and capillaries; NL in lumen of bronchioles and alveoli (partly degen) and in association with degen BEC | 15.12 (13.47) | 29.7 | 3.69 |
| **5 x** **10^5 TCID50, 2 dpi**  [1.1.2] | **HE:** bronchioles with abundant degen EC (in place and sloughed off); parenchymal areas with type II pn activation, occ degen AEC and increased cellularity  **vAg:** extensive expression in bronchus/bronchioles (almost all BEC, some also degen); most alveoli with pos AEC  **Ly6G:** increase in NL in lumen of vessels and capillaries, NL in lumen of bronchioles and alveoli (partly degen) and in association with degen BEC | 15.92 (13.79) | 24.5 | 4.26 |
| **5 x** **10^5 TCID50, 2 dpi**  [1.1.3] | **HE:** bronchioles with abundant degen EC (in place and sloughed off); parenchymal areas with type II pn activation, occ degen AEC and increased cellularity  **vAg:** extensive expression in bronchus/bronchioles (almost all BEC, some also degen); most alveoli with pos AEC  **Ly6G:** increase in NL in lumen of vessels and capillaries, NL in lumen of bronchioles and alveoli (partly degen) and in association with degen BEC | 15.56 (13.83) | 22.6 | 4.31 |
| **5 x** **10^5 TCID50, 2 dpi**  [1.1.4] | **HE:** bronchioles with abundant degen EC (in place and sloughed off); parenchymal areas with type II pn activation, occ degen AEC and increased cellularity  **vAg:** extensive expression in bronchus/bronchioles (almost all BEC, some also degen); most alveoli with pos AEC  **Ly6G:** increase in NL in lumen of vessels and capillaries, NL in lumen of bronchioles and alveoli (partly degen) | 16.33 (14.81) | 20.8 | 4.69 |
|  |  |  |  |  |
| **5 x** **10^5 TCID50, 4 dpi**  [1.2.1] | **HE:** one bronchiole with degen EC (in place and sloughed off), others unaltered; large parenchymal area with extensive type II pn activation, degen AEC and increased cellularity (leukocytes incl NL)  **vAg:** some bronchioles with extensive expression (almost all BEC, some also degen), others with a few individual or no pos BEC; patches of alveoli with pos AEC (within and outside affected areas)  **Ly6G:** some increase in NL in lumen of vessels and capillaries, NL in lumen of bronchioles and alveoli in affected area (partly degen) | 31.68 (11.64) | 3.5 | 1.65 |
| **5 x** **10^5 TCID50, 4 dpi**  [1.2.2] | **HE:** bronchioles with a few degen EC (in place or sloughed off), others unaltered; small parenchymal area with type II pn activation, degen AEC and increased cellularity (leukocytes incl NL)  **vAg:** some bronchioles with several (partly degen) pos BEC, most with a few individual or no pos BEC; patches of alveoli with pos AEC (within and outside affected area)  **Ly6G:** some increase in NL in lumen of vessels and capillaries, a few NL in lumen of bronchioles and alveoli in affected area (partly degen) | 31.86 (11.78) | 7.2 | 1.74 |
| **5 x** **10^5 TCID50, 4 dpi**  [1.2.3] | **HE:** bronchioles with a few degen EC (in place or sloughed off), others unaltered; small parenchymal area with type II pn activation, degen AEC and increased cellularity (leukocytes incl NL)  **vAg:** some bronchioles with several (partly degen) pos BEC, most with a few individual or no pos BEC; patches of alveoli with pos AEC (within and outside affected area)  **Ly6G:** some increase in NL in lumen of vessels and capillaries, a few NL in lumen of bronchioles and alveoli in affected area (partly degen) | 31.33 (11.71) | 5.2 | 1.95 |
| **5 x** **10^5 TCID50, 4 dpi**  [1.2.4] | **HE:** bronchioles with rare degen EC, most unaltered; parenchymal area with type II pn activation, degen AEC and increased cellularity (leukocytes incl NL)  **vAg:** some bronchioles with a few to several (partly degen) pos BEC, most with a few individual or no pos BEC; patches of alveoli with pos AEC (within and outside affected area)  **Ly6G:** some increase in NL in lumen of vessels and capillaries, a few NL in lumen of bronchioles and alveoli in affected area (partly degen) | 35.02 (11.89) | 2.2 | 1.57 |
|  |  |  |  |  |
| **PBS (control)**  [1.3.1] | **Lung:** NHA  **vAg:** neg  **Ly6G:** a few individual NL in lumen of vessels and capillaries | n.d., set to 40 (13.65) | n.d. | 0.45 |
| **PBS (control)**  [1.3.2] | **Lung:** NHA  **vAg:** neg  **Ly6G:** a few individual NL in lumen of vessels and capillaries | n.d., set to 40 (15.66) | n.d. | 0.39 |
| **PBS (control)**  [1.3.3] | **Lung:** NHA  **vAg:** neg  **Ly6G:** a few individual NL in lumen of vessels and capillaries | n.d., set to 40 (14.32) | n.d. | 0.41 |
| **PBS (control)**  [1.3.4] | **Lung:** NHA  **vAg:** neg  **Ly6G:** a few individual NL in lumen of vessels and capillaries | n.d., set to 40 (14.57) | n.d. | 0.34 |

**B. Experiment 2.** Infected animals were euthanized and Ly6G neutrophils isolated from lungs at 2 and 4 days post infection.

| **Infection, dpi**  [Animal no] |
| --- |
| **5 x** **10^5 TCID50, 2 dpi**  [2.1.1] |
| **5 x** **10^5 TCID50, 2 dpi**  [2.1.2] |
| **5 x** **10^5 TCID50, 2 dpi**  [2.1.3] |
| **5 x** **10^5 TCID50, 2 dpi**  [2.1.4] |
|  |
| **5 x** **10^5 TCID50, 4 dpi**  [2.2.1] |
| **5 x** **10^5 TCID50, 4 dpi**  [2.2.2] |
| **5 x** **10^5 TCID50, 4 dpi**  [2.2.3] |
| **5 x** **10^5 TCID50, 4 dpi**  [2.2.4] |
|  |
| **PBS (control), 2 dpi**  [2..3.1] |
| **PBS (control), 2 dpi**  [2.3.2] |
| **PBS (control), 2 dpi**  [2.3.3] |
| **PBS (control), 2 dpi**  [2.3.4] |

**C.** **Experiment 3**. Infected animals were treated with control isotype or IFNAR blocking antibody prior to euthanization and examination at 2 days post infection.

| **Infection, treatment**  [Animal no] | **Histological changes, viral antigen (vAg), Ly6G and histone H3 expression^1^ in lungs** | **Viral subE (and GAPDH) RNA Cts^2^** | **Viral antigen expression (% lung area)^3^** | **Ly-6G neutrophils (% lung area)^3^** |
| --- | --- | --- | --- | --- |
| **5 x** **10^5 TCID50, control isotype**  [3.1.1] | **HE:** bronchus and connected bronchiole with abundant degen EC (in place or sloughed off); endothelial cell activation in adjacent muscular veins, with some leukocyte adhesion; adjacent parenchymal areas with type II pn activation, occ degen AEC and increased cellularity  **vAg:** extensive expression in bronchus/bronchioles (almost all BEC, also degen), and large adjacent areas of alveoli with pos AEC  **Ly6G:** increase in NL in lumen of vessels and capillaries in affected areas, NL in lumen of bronchioles and alveoli (partly degen)  **H3:** pos reaction in areas with degen NL (lumen of bronchioles, affected alveoli) | 14.75 (12.63) | 13.1 | 0.57 |
| **5 x** **10^5 TCID50, control isotype**  [3.1.2] | **HE:** some bronchioles with abundant degen EC (in place or sloughed off); endothelial cell activation in some adjacent vessels; (adjacent) parenchymal areas with type II pn activation, occ degen AEC and increased cellularity  **vAg:** extensive expression in some bronchioles (almost all BEC, also degen), large (adjacent) areas of alveoli with pos AEC  **Ly6G:** mild increase in NL in lumen of vessels and capillaries in affected areas, NL in lumen of bronchioles and alveoli (partly degen)  **H3:** pos reaction in areas with degen NL (lumen of bronchioles, affected alveoli) | 16.61 (11.54) | 19.5 | 0.54 |
| **5 x** **10^5 TCID50, control isotype**  [3.1.3] | **HE:** bronchioles with abundant degen EC (in place or sloughed off); endothelial cell activation in some muscular veins, with leukocyte rolling and subendothelial infiltration (vasculitis), other vessels packed with leukocytes; (adjacent) parenchymal areas with type II pn activation, occ degen AEC and increased cellularity  **vAg:** extensive expression in bronchioles (patches of BEC to almost all BEC, also degen), large (adjacent) areas of alveoli with pos AEC  **Ly6G:** mild increase in NL in lumen of vessels and capillaries in affected areas, NL in lumen of bronchioles and alveoli (often degen)  **H3:** extensive pos reaction in areas with degen NL (lumen of bronchioles, affected alveoli) | 16.00 (12.71) | 24.3 | 1.64 |
| **5 x** **10^5 TCID50, control isotype**  [3.1.4] | **HE:** bronchus and bronchioles with abundant degen EC (in place or sloughed off); endothelial cell activation in some muscular veins, with leukocyte rolling and pv infiltration; (adjacent) parenchymal areas with type II pn activation, occ degen AEC and increased cellularity  **vAg:** extensive expression in bronchioles (patches to almost all BEC, also degenerate), large (adjacent) areas of alveoli with pos AEC  **Ly6G:** mild increase in NL in lumen of vessels and capillaries in affected areas, NL in lumen of bronchioles and alveoli (partly degenerate)  **H3:** pos reaction in areas with degen NL (lumen of bronchioles, affected alveoli); most extensive in alveoli | 15.07 (13.83) | 15.3 | 0.28 |
|  |  |  |  |  |
| **5 x** **10^5 TCID50,**  **anti-IFNAR**  [3.2.1] | **HE:** bronchus and connected bronchiole with abundant degen EC (in place or sloughed off); endothelial cell activation in muscular veins, with some leukocyte adhesion; patchy parenchymal areas with type II pn activation, occ degen AEC and increased cellularity  **vAg:** extensive expression in bronchioles (abundant to almost all BEC, also degen), and large areas of alveoli with pos AEC  **Ly6G:** increase in NL in lumen of vessels and capillaries in affected areas, NL in lumen of bronchioles and alveoli (partly degen)  **H3:** pos reaction in areas with degen NL (lumen of bronchioles, affected alveoli) | 13.98 (12.44) | 23.0 | 1.13 |
| **5 x** **10^5 TCID50,**  **anti-IFNAR**  [3.2.2] | **HE:** bronchioles with occ degen EC (in place or sloughed off); endothelial cell activation in muscular veins, with some leukocyte adhesion; parenchymal areas with type II pn activation, occ degen AEC and increased cellularity  **vAg:** extensive expression in bronchus/bronchioles (almost all BEC, also degen), and large areas of alveoli with pos AEC  **Ly6G:** increase in NL in lumen of vessels and capillaries in affected areas, NL in lumen of bronchioles and alveoli (partly degen)  **H3:** pos reaction in areas with degen NL (lumen of bronchioles, affected alveoli) | 14.90 (13.12) | 25.8 | 0.89 |
| **5 x** **10^5 TCID50,**  **anti-IFNAR**  [3.2.3] | **HE:** infected bronchus with a few degen EC (in place); mild endothelial cell activation in some adjacent vessels; (adjacent) parenchymal areas with type II pn activation and increased cellularity  **vAg:** a few individual EC in bronchus and some bronchioles, one with all EC pos, adjacent large patches of alveoli with pos AEC  **Ly6G:** increase in individual NL in lumen of vessels and capillaries (mainly in affected areas), a few NL in lumen and between EC of infected bronchiole, and in alveoli in affected areas  **H3:** rare pos reaction in alveoli | 28.79 (14.09) | 2.7 | 0.08 |
| **5 x** **10^5 TCID50,**  **anti-IFNAR**  [3.2.4] | **HE:** infected bronchioles with variable amount of degen EC (in place or sloughed off); endothelial cell activation in some vessels, with leukocyte rolling and pv infiltration; (adjacent) parenchymal areas with type II pn activation, occ degen AEC and increased cellularity  **vAg:** bronchus and bronchioles with individual, patches of to almost all BEC pos (also degen) and cell free viral Ag in lumen, large (adjacent) areas of alveoli with pos AEC (also degen)  **Ly6G:** increase in NL in lumen of vessels and capillaries in affected areas, NL in lumen of bronchioles and alveoli (partly degen)  **H3:** extensive pos reaction in areas with degen NL (lumen of bronchioles, affected alveoli) | 13.54 (13.33) | 22.3 | 1.66 |
|  |  |  |  |  |
| **PBS (control)**  [3.3.1] | **Lung:** NHA  **vAg:** neg  **Ly6G:** a few individual NL in lumen of vessels and capillaries | n.d., set to 40 (10.20) | 0.0 | 0.14 |
| **PBS (control)**  [3.3.2] | **Lung:** NHA  **vAg:** neg  **Ly6G:** a few individual NL in lumen of vessels and capillaries | n.d., set to 40 (11.04) | 0.0 | 0.09 |
| **PBS (control)**  [3.3.3] | **Lung:** NHA  **vAg:** neg  **Ly6G:** a few individual NL in lumen of vessels and capillaries | n.d., set to 40 (14.06) | 0.0 | 0.15 |
| **PBS (control)**  [3.3.4] | **Lung:** NHA  **vAg:** neg  **Ly6G:** a few individual NL in lumen of vessels and capillaries | n.d., set to 40 (13.70) | 0.0 | 0.10 |

**C.** **Experiment 4**. Infected animals were euthanized and examined at 2 and 4 days post infection or treated with control isotype or IFNAR blocking antibody prior to euthanization at 2 days post infection.

| **Infection, treatment**  [Animal no] | **Viral subE (and GAPDH) RNA Cts^2^** | **Infectious virus titer (FFU/lung, log10)** | **Viral antigen expression (% lung area)^3^** | **Ly-6G neutrophils (% lung area)^3^** | **Ly-6G neutrophil/lymphocyte ratio^4^** | **Ly-6G neutrophil count (10^5^/lung)^4^** |
| --- | --- | --- | --- | --- | --- | --- |
| **5 x** **10^5 TCID50, 2 dpi**  [4.1.1] | 13.29 (11.13) | 9 | 13.0 | 0.6 | 0.34 | 26.9 |
| **5 x** **10^5 TCID50, 2 dpi**  [4.1.2] | 16.55 (14.63) | 8 | 13.3 | 1.6 | 0.26 | 27.3 |
| **5 x** **10^5 TCID50, 2 dpi**  [4.1.3] | 17.17 (11.48) | 9 | 14.5 | 3.1 | 0.29 | 28.2 |
| **5 x** **10^5 TCID50, 2 dpi**  [4.1.4] | 20.51 (16.98) | 9 | 13.6 | 2.7 | 0.26 | 54.1 |
|  |  |  |  |  |  |  |
| **5 x** **10^5 TCID50, 4 dpi**  [4.2.1] | 21.19 (10.55) | 6 | 6.7 | 0.4 | 0.35 | 23.6 |
| **5 x** **10^5 TCID50, 4 dpi**  [4.2.2] | 19.35 (12.57) | 6 | 6.8 | 1.1 | 0.21 | 18.3 |
| **5 x** **10^5 TCID50, 4 dpi**  [4.2.3] | no Ct, set to 40 (11.80) | 5 | 2.0 | 0.9 | 0.27 | 27.7 |
| **5 x** **10^5 TCID50, 4 dpi**  [4.2.4] | 21.87 (11.65) | 5 | 2.7 | 1.8 | 0.19 | 38.7 |
|  |  |  |  |  |  |  |
| **PBS (control)**  [4.3.1] | n.d., set to 40 (14.60) | n.d. | 0.1 | 0.2 | 0.15 | 17.4 |
| **PBS (control)**  [4.3.2] | n.d., set to 40 (15.03) | n.d. | 0.4 | 0.2 | 0.14 | 16.6 |
| **PBS (control)**  [4.3.3] | n.d., set to 40 (13.02) | n.d. | 0.6 | 0.3 | 0.12 | 17.1 |
| **PBS (control)**  [4.3.4] | n.d., set to 40 (19.93) | n.d. | 0.1 | 0.3 | 0.08 | 8.6 |
|  |  |  |  |  |  |  |
| **5 x** **10^5 TCID50, control isotype**  [4.4.1] | 14.65 (11.43) | 9 | 22.6 | 2.9 | 0.27 | 41.0 |
| **5 x** **10^5 TCID50, control isotype**  [4.4.2] | 15.59 (15.00) | 10 | 23.3 | 1.6 | 0.21 | 31.0 |
| **5 x** **10^5 TCID50, control isotype**  [4.4.3] | 15.33 (12.93) | 6 | 10.8 | 1.6 | 0.30 | 27.7 |
| **5 x** **10^5 TCID50, control isotype**  [4.4.4] | 17.08 (13.55) | 8 | 12.6 | 2.8 | 0.29 | 38.9 |
|  |  |  |  |  |  |  |
| **5 x** **10^5 TCID50,**  **anti-IFNAR**  [4.5.1] | 11.94 (11.49) | 8 | 26.6 | 4.4 | 0.61 | 77.6 |
| **5 x** **10^5 TCID50,**  **anti-IFNAR**  [4.5.2] | 13.44 (12.81) | 7 | 0.0 | 1.0 | 0.33 | 50.4 |
| **5 x** **10^5 TCID50,**  **anti-IFNAR**  [4.5.3] | 14.19 (13.34) | 8 | 33.8 | 1.9 | 0.40 | 55.2 |
| **5 x** **10^5 TCID50,**  **anti-IFNAR**  [4.5.4] | 14.91 (13.23) | 8 | 19.8 | 1.2 | 0.26 | 42.6 |

The age of the infected animals were 9 weeks  in experiment 1 and 8 weeks in experiments 2,3 and 4; alv – alveolar; BEC – bronchiolar epithelial cells; degen – degenerate (this also includes apoptotic and/or necrotic); EC – epithelial cells; F – female; HE – histological features assessed in a hematoxylin-eosin stained section; incl – including; M – male; neg – negative; NHA – no histological abnormality; NL – neutrophilic leukocytes (i.e. neutrophils); occ – occasional; pc – pneumocytes; pos – positive; pv – perivascular; vAg – viral antigen; we – weeks; n.d. – not detected

^1^Viral antigen and Ly6G expression determined by immunohistochemistry.

^2^Obtained RT-qPCR cycle threshold values (Cts) for viral subgenomic E gene (subE) as a measure of viral replication and GADPH mRNA as the housekeeping gene. As expected, for PBS control animals no Ct value for viral subE was obtained. The subE Ct for control animals was set to the detection limit of 40 to facilitate fold change calculations presented in Figure 7A and Supplementary Fig. 7B.

^3^Viral antigen and Ly-6G expression per % lung area calculated by morphometry after immunohistochemical staining of lung tissue.

^4^Neutrophil /lymphocyte ratio and total neutrophil counts in lung single cell suspensions assessed by flow cytometry.
