# Supplementary material for: The assembly of neutrophil inflammasomes during COVID-19 is mediated by type I interferons
Source: PLoS Pathog. 2024 Aug 22;20(8):e1012368. doi: 10.1371/journal.ppat.1012368 (PMC11340896; doi:10.1371/journal.ppat.1012368)
Supplement: Attachment — Submitted filename: 140933_1_rebuttal_2921587_s82zth.pdf [file ppat.1012368.s012.pdf]

Revision Plan

Review COMMONS

Manuscript number: RC-2023-02204R
Corresponding author(s): Luz Eneida, Cabrera

[The "revision plan" should delineate the revisions that authors intend to carry out in response to the points raised by the referees. It also provides the authors with the opportunity to explain their view of the paper and of the referee reports.

The document is important for the editors of affiliate journals when they make a first decision on the transferred manuscript. It will also be useful to readers of the reprint and help them to obtain a balanced view of the paper.

If you wish to submit a full revision, please use our "Full Revision" template. ***It is important to use the appropriate template to clearly inform the editors of your intentions.****]*

1. General Statements [optional]

Response*: We would like to thank both reviewers for their insightful comments. We have addressed most comments in the transferred manuscript and are willing to perform additional experiments to respond to some remaining points, as detailed in the following sections.*

2. Description of the planned revisions

Insert here a point-by-point reply that explains what revisions, additional experimentations and analyses are planned to address the points raised by the referees.

Reviewer #1:

Fig. 5, It would also be interesting to explore the involvement of IFN-I receptors (IFNAR1 vs IFNAR2) by dissecting IFN-α from IFN-β responses.

Response*: Our understanding is that IFNAR1 and IFNAR2 form a heterodimer, which can be activated by both IFN-α and IFN-β. It is thus difficult to dissect the role of individual receptors or cytokines from each other. However, to confirm that IFN-α and IFN-β are both acting through the IFNAR1 receptor to prime for IL-1b activation and release in human neutrophils, we will perform additional experiments by treating neutrophils isolated from healthy individuals with both cytokines individually (as opposed to using them synergistically as in the current manuscript) and blocking their effects by a commercial IFNAR1 blocking antibody.*

Reviewer #2:

- The authors are encouraged to specify the number of independent experiments conducted. For mouse model studies, it is recommended to include results that are

either representative of, or aggregated from, a minimum of two independent experiments to ensure robustness of the data.

Response: *We did three independent animal experiments in total. The first two experiments involved two time points (2 and 4 days post infection) with different downstream experiments: The first experiment was used for the detection of viral loads by PCR as well as IHC-based quantification of viral antigen and neutrophil influx into the lungs (Exp1, new suppl. Fig 6) while the second experiment (Exp2) was used to isolate neutrophils from the lungs in order to assess neutrophil caspase1 activity and to obtain samples for RNAseq (new Fig.7 A-D). The third experiment (Exp3) was to perform anti-IFNAR and isotype treatments for infected mice; here, all animals were at day 2 post infection. For this experiment we prepared lung samples for IHC and PCR (new suppl Fig. 7) and isolated neutrophils for the assessment of caspase1 activity and for PCR assays (new Fig. 7E-G). We have now improved the text and supplementary Table S2 to indicate these separate experiments more clearly. In our experience, this approach, i.e. making use of the lungs of oe animals for the determination of several parameters, is of benefit not only because we can directly compare these parameters in a given animal and can thereby reduce the number of animals used for the project (following the 3R principles).*

However, in order to increase sample size and to respond to the various issues raised by the reviewer regarding our mouse work, we have decided to undertake another mouse infection experiment to analyze the effect of the anti-IFNAR treatment on viral loads by assaying for viral titers in the lung sample (which will hopefully directly answer the reviewer's concerns raised below regarding the effect of anti-IFNAR treatment on viral replication). We will also confirm the negative effect of anti-IFNAR treatment on the expression of IFN-responsive genes by measuring OAS2 mRNA levels by PCR in the lungs of the anti-IFNAR treated as compared to isotype-treated mice. Finally, we will isolate neutrophils from the lungs to repeat the experiment showing the effect of anti-IFNAR treatment on neutrophil inflammasome activity as shown in Fig 7. E-G and will take a sample each for the histological and immunohistological analysis to complement the other tests.

-The authors are advised to employ more quantitative methods, such as flow cytometry, to measure neutrophil recruitment in mice. Additionally, it should be clarified how many tissue sections from each mouse were assessed for every experimental condition to ensure the reproducibility and statistical validity of the results.

Response: *While we are not aware that flow cytometry can be considered as a "more quantitative method" than morphometry, we agree that it is an alternative, i.e. complementary quantitative method. Furthermore, we feel that direct quantification of isolated Ly-6G+ neutrophils which are obtained from homogenized lung tissue by magnetic beads (as we have done for some mouse experiments of the current manuscript) is another quantitative approach and a method comparable to flow cytometry. Therefore, we are willing to repeat the mouse infection experiments and quantify the isolated neutrophils in parallel to IHC-based morphometry in order to determine the robustness of our morphometrical neutrophil quantification but are not inclined to undertake flow cytometry, in particular since this would not allow the assessment of all other relevant parameters in the same lung.*

The reviewer asked for information regarding the number of tissue sections that were assessed from each mouse. In our opinion, for quantitative purposes (i.e. morphometry), it is more meaningful to determine the total tissue area that is examined, as different pathologists take different approaches to trim the lung for histological examination (cross sections vs. longitudinal section of a lobe). In our study we examined a tissue area of 19.5 ± 6 mm^2 for each lung, which is stated now also in the revised manuscript.

Revised manuscript, methods section, line 307:

"The average total tissue area used for the quantification was 19.5 ± 6 mm^2".

-The authors need to address discrepancies in their text regarding the effects of anti-IFNAR1 blockade on viral titers and neutrophil recruitment in SARS-CoV-2 infected mice. While they state there is no change, Supplementary Figure 5D suggests increased SARS-CoV-2 NP staining with anti-IFNAR1 treatment, and there appears to be a lack of quantitative data on lung neutrophils to substantiate the claim that neutrophil recruitment remains unaffected. It is necessary for the authors to provide a more detailed explanation or additional data to resolve these inconsistencies

***Response**: We did not observe any significant differences in the antigen expression between anti-IFNAR treated and isotype-treated mice. The lack of differences in viral loads was also shown by PCR (suppl Fig. 6B). However, since we are suggesting to repeat the anti-IFNAR experiment which will also include the quantification of viral titers in lung tissue with and without anti-IFNAR treatment, we will gain further insight whether and how IFN-I activity could regulate infection kinetics. We will also repeat the quantification of neutrophils in the lungs of anti-IFNAR and isotype-treated mice by IHC-based morphometry as well as by determining the number of isolated LY-6G+ neutrophils.*

- The authors should demonstrate the effectiveness of anti-IFNAR1 blockade in mice by providing evidence of sustained inhibition of IFN-I signaling throughout the duration of the experiment to validate the treatment protocol used.

***Response:** We feel the observed inhibition of inflammasome-related pathways by anti-IFNAR treatment strongly argues that blockade of IFNAR activity was successful during the 2 day time course of the direct experiment (Exp3). However, the reviewer's comment is valid since we have not shown the effect of anti-IFNAR treatment on specific IFN-induced genes. Therefore, we will repeat the anti-IFNAR treatment in infected mice and confirm its negative effect on the expression of IFN-responsive genes by detecting the expression of OAS2 mRNA in lung samples by PCR (OAS2 is one of the mostly upregulated genes by SARS-CoV-2 infection based on our neutrophil transcriptomics analysis). We will also assess whether the treatment directly affects the levels of infectious virus by quantifying viral titers in lung tissue.*

*****Referee Cross-Commenting*****

-I agree with Reviewer#1 about trying to dissect the role of IFNAR-1 vs IFNAR-2. Authors

partially look at this in the in vivo mice experiments using the anti-IFNAR1 blocking Ab, but it would reinforce the study to see if this holds with human cells.

Response: *To confirm that IFN-α and IFN-β are both acting through the IFNAR1 receptor to prime for IL-1b activation in human neutrophils, we will perform additional experiments by treating neutrophils isolated from healthy individuals with both cytokines individually (as opposed to using them synergistically as done for the current manuscript) and blocking their effects by a commercial IFNAR1 blocking antibody.*

The study presents an investigation into the role of neutrophils and inflammasome formation in COVID-19 pathology, contributing to the field with transcriptomic profiling of neutrophils from varying severity of patient cases and a SARS-CoV-2 mouse model. A significant IFN-I gene signature in severe cases was confirmed, and differences in inflammasome response were identified, adding to our understanding of disease mechanisms.

Strengths of the paper include the comprehensive analysis of neutrophil maturation states and the novel insights into the priming of inflammasome activation by IFN-I. However, limitations were noted in the purity of samples for RNAseq analysis and the lack of conclusive in vivo evidence for the direct role of IFN-I in neutrophil inflammasome priming. The study's implications suggest potential avenues for targeted therapies, but the authors were advised to moderate their conclusions without stronger in vivo evidence and to clarify the potential therapeutic implications of their findings.

Additional suggestions for improvement include the use of more quantitative methods like flow cytometry for neutrophil recruitment measurements, clarification on experimental replication, and resolving discrepancies in data presentation regarding anti-IFNAR1 blockade effects. Furthermore, the paper would benefit from discussing the relevance of autoantibodies against IFN-I in the context of their findings and from exploring the causal relationship between inflammasome patterns and disease severity.

Response: *We would like to thank the reviewer for highly impactful comments. As suggested, we have redone the RNAseq analysis including only samples of higher neutrophil content, with similar conclusions as those previously made, and have amended the discussion based on the reviewer's comments. Furthermore, to address the remaining questions raised by the reviewer, we will undertake the following additional animal experiments:*

- *Five groups of animals (n = 4);*
    - *PBS-inoculated animals*
    - *Infected with SARS-CoV-2 for 2 days, treated with isotype*
    - *Infected with SARS-CoV-2 for 2 days, treated with anti-IFNAR*
    - *Infected with SARS-CoV-2 for 2 days*
    - *Infected with SARS-CoV-2 for 4 days*
    - *Measurements from lung tissue*

- *Quantification of LY-6G neutrophils by morphometry*
- *RT-PCR for OAS2 mRNA (assessment of the successful blockade of IFN-I signaling by IFNAR antibody)*
- *Viral titers by quantification of infectious virus in cell culture (assessment of the successful blockade of IFN-I signaling by IFNAR antibody)*
- *Isolation of LY-6G neutrophils from lung tissue*
  - *Quantification of the number of isolated LY-6G neutrophils*
  - *Repeating measurement of caspase1 activity*
  - *Repeating RT-PCR for caspase1 and IL-1b mRNA*

3. Description of the revisions that have already been incorporated in the transferred manuscript

Please insert a point-by-point reply describing the revisions that were already carried out and included in the transferred manuscript. If no revisions have been carried out yet, please leave this section empty.

Reviewer #1:

IFN-I promotes immune responses to diverse viruses. Some of them have evolved to dampen IFN-I responses in order to weaken or delay antiviral responses. A number of studies support the idea that this could be the case of SARS-CoV-2 (0.1038/s41586-022-04447-0, 10.1016/j.it.2021.02.003, 10.1038/s41467-022-34895-1, to cite a few). The authors should refer to some of these studies to counterbalance the statements of increased IFN-I response in severe COVID-19 patients in some parts of the manuscript, such as in the Discussion.

***Response**: This is a good point and we added discussion on this topic.*

Revised manuscript, discussion section, between lines 634-648:

"It should be noted that several SARS-CoV-2 encoded proteins have been shown to inhibit IFN-I signaling (60). However, no evidence suggests that neutrophils can be infected by SARS-CoV-2 and therefore it seems unlikely that such direct virus mediated effects could play a role in the observed neutrophil unresponsiveness to IFN-I.

The dualistic nature of the IFN-I response in COVID-19 has been recognized previously. It seems that a strong initial IFN-I response to SARS-CoV-2 is more likely to result in asymptomatic or mild COVID-19 whereas a decreased initial IFN-I activity, due to e.g. genetic defects or increased levels of IFN-I autoantibodies, can lead to more severe COVID-19 (61). This initial beneficial effect of IFN-I is probably due to its ability to limit viral replication at early stages of the infection. However, at later stages of the disease IFN-I can be detrimental by

promoting inflammatory pathways instead of direct antiviral effects (62). Thus, similarly to the IFN-I response in general, the role of neutrophil inflammasomes in development and severity of COVID-19 might be dualistic in nature with an initial protective effect while damaging when sustained for prolonged periods."

60. Rashid F, et al. Roles and functions of SARS-CoV-2 proteins in host immune evasion [preprint]. Front Immunol. 2022;13. https://doi.org/10.3389/fimmu.2022.940756.
61. da Silva RP, et al. Circulating Type I Interferon Levels and COVID-19 Severity: A Systematic Review and Meta-Analysis. Front Immunol. 2021;12. https://doi.org/10.3389/fimmu.2021.657363.
62. Smith N, et al. Defective activation and regulation of type I interferon immunity is associated with increasing COVID-19 severity. Nat Commun. 2022;13(1). https://doi.org/10.1038/s41467-022-34895-1.

In Fig 5, the authors stimulate PMNs from healthy controls in vitro with IFN-I and use mainly IL-1b as a readout for neutrophil priming. The authors should analyse whether IFN-I-mediated priming ultimately leads to NETosis, given the relevance of NETs to COVID-19 pathology (10.1016/j.tips.2023.06.007), which is acknowledged by the authors in the Introduction.

***Response**: We agree that NETosis is a potential outcome of neutrophil activation. This is in part why we assayed for the release of MPO, which can be used as a marker of NETosis, in the ex vivo activated PMNs. However, while a clear increase in MPO levels was observed in response to nigericin treatment, no increase was seen with IFN-I or LPS treatments alone in COVID-19 or HC PMNs (Fig. 4C). We slightly modified the text to make it clearer that MPO served to detect PMN degranulation and NETosis in our study.*

Revised manuscript, results section, lines 440-444:

"However, the release of myeloperoxidase (MPO), used as a marker of degranulation and/or NETosis, in response to nigericin was similar between COVID-19 PMNs and HC PMNs, and therefore the observed diminished IL-1β release by COVID-19 PMNs is not due to general cellular inertia but may be specific to the ex vivo induced inflammasome pathway."

In Methods (Histology and immunohistochemistry), the authors mention that histone H3 is a NET marker and use this in Supp Fig 5 to support evidence of NETosis. The authors should state which antibody clone/company was used. Histone H3 is expressed in high levels by non-NETotic neutrophils. Citrullinated histone H3 (CitH3), on the other hand, is detectable by few commercially available antibodies and can be used as a NET marker in conjunction with other markers such as DNA staining (10.1084/jem.20201129).

***Response**: For immunohistochemical detection of NETosis, we have used the following antibody: rabbit anti-histone H3 (citrulline R2 + R8 + R17; Abcam). We have provided a reference to a publication of a co-author (Schmid AS, et al. Antibody-based targeted delivery of*

interleukin-4 synergizes with dexamethasone for the reduction of inflammation in arthritis. Rheumatology (United Kingdom). 2018;57(4):748–755. Doi: 10.1093/rheumatology/kex447) which includes this information and fully describes the staining protocol. We admit that our labelling of the figure and the text was misleading by stating that the used antibody was anti-histone H3 instead of indicating that an antibody detecting citrullinated form of histone H3 was used. We have therefore relabeled Supplementary Fig. 7 and rewritten the text to indicate this more clearly.

In the publication kindly referred to by the reviewer, the following antibody was used: rabbit anti-histone H3 (H3Cit; Abcam; cat. ab5103; 1:500) in immunofluorescence, where DAPI fluorescence served to highlight the DNA. We generally work with immunohistochemistry instead of immunofluorescence as it allows better alignment with histopathological features. However, we have now applied the anti-H3cit antibody in a fluorescence protocol, using the same tissues and antibody as shown in the immunohistochemistry image of suppl. Fig7, to indicate that the used antibody works equally well in immunofluorescence and immunohistochemistry.

Below is a figure plate that shows: NET IHC, showing abundant expression in the lumen of a bronchiole (top) and NET IF, showing part of a bronchiole with NET (green) and nuclei (DAPI; blue) and a closer view (bottom) with NET expression in a cell with the nuclear morphology of a neutrophil (arrowhead).

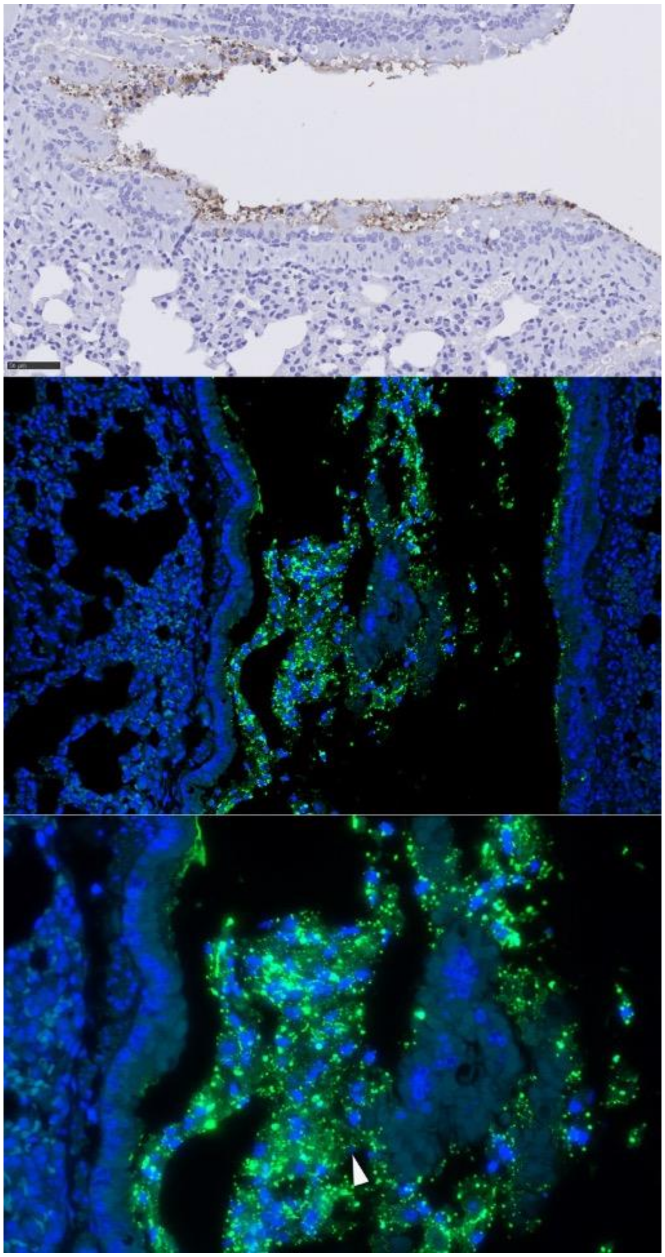

In Fig 8, the authors show that neutrophils migrate to the lungs of infected animals. This finding is showed in many previous studies and does not seem to favour the structure of the manuscript. Insteand, it seems it would fit a Supp Fig better, or a portion of the following figure.

***Response**: We are aware that neutrophil recruitment into the lungs with SARS-CoV-2 infection has been shown previously. However, this was so far not done in the model that we have used,*

mouse-adapted SARS-CoV-2 infection in wild type (BALB/C) mice in which the infection is short-lived and wanes off after 4 days (Gawish et al., 2022). We are happy to move this figure plate to the Supplements as Reviewer #2 shares this opinion. In the revised manuscript, it now features as Supplementary Fig. 6.

Reviewer #2:

Major points:

-Figure 2A: for this RNAseq analysis, the authors claim that "This analysis also demonstrated that cells in the LDG fraction were predominantly immature neutrophils, meanwhile PMNs were composed of mainly mature neutrophils (Figure 2A)". Nevertheless, there are 2 samples in the "Severe COVID-19" group that show a fraction of neutrophils {less than or equal to}0.6, which indicates low levels of purity. In addition, there is one "PMN" sample with high LDG fraction (around 40%). Authors should remove from this analysis samples with such a low purity since the big fraction ({greater than or equal to}40%) of contaminating cells could introduce a bias in this group. Are the differences observed still present in the absence of these low-purity samples?

Response: *For Figure1 of the original manuscript, we first performed the analyses including all the samples, and after assessing their purity, included only the samples with highest purity in the following figures. However, we followed the reviewer's recommendation and redefined the purity of the samples as ≥65% of total neutrophils, independent of their maturity. Of note, unlike the reviewer suggests, we did not remove the one "PMN" sample with high degree of immaturity, since PMNs and LDGs are defined based on their isolation method and not their degree of maturity. Along these lines, we have decided to move the new unsupervised heatmap and present it together with the RNA deconvolution plot as supplementary Figure 1. Therefore, Figure 1 now contains the samples with high purity based on the redefined criteria, with PCA in panel A, pathway analyses in panel B (PMN vs LDG) and interferon-related genes heatmap in panel C. We also redid suppl Fig. 2 to include samples with the redefined criteria and modified the results text accordingly.*

Revised manuscript, results section, lines 333-380:
"Unsupervised RNA-seq analysis reveals an antiviral gene expression signature of circulating neutrophils in COVID-19 that is strongly influenced by maturity

With our recent findings on increased frequencies of low-density granulocytes (LDGs, isolated from the PBMC fraction) during COVID-19 and their likely relevant role in disease progression (7), we sought to understand in more detail how the transcriptomic profile of LDGs differs from their higher "normal" density counterpart, the circulating polymorphonuclear cells (PMNs) (31), typically consisting mainly of mature neutrophils. Neutrophils isolated from different cohorts comprised three PMN groups (severe COVID-19, mild COVID-19, and healthy controls), and one LDG group. Initial deconvolution of the RNA sequencing (RNA-seq) data allowed us to gain a comprehensive understanding of the cellular composition within PMN and LDG fractions and verified that most cells present in the samples were neutrophils (Supplementary Figure 1A).

This analysis also demonstrated that cells in the LDG fraction were predominantly immature neutrophils, meanwhile PMNs were composed of mainly mature neutrophils.

The samples with predominant neutrophil cell populations were selected for subsequent gene expression analysis (neutrophils ≥ 65 %). The high variance in gene expression between PMNs and LDGs was confirmed by principal component analysis (PCA) (Figure 1A), which revealed that the gene expression patterns of COVID-19 LDGs differed from those of all PMNs regardless of the patients' disease state. Functional enrichment analyses through gene overrepresentation (ORA) and gene-set enrichment analyses (GSEA) (Figure 1B) compared PMNs with LDGs from severe COVID-19 patients. The most statistically significant result was an overrepresentation of the NOD-like receptor signaling pathway in PMNs in contrast with LDGs, highlighting that the different neutrophil fractions have a distinct inflammatory profile. This was supported by GSEA, where the most obvious increases in fold changes were the enrichment of the interferon signaling pathways. Another relevant difference was the cell cycle and DNA replication pathways, identified by both ORA and GSEA, which supported our previous findings suggesting LDGs to be predominantly immature cells (7). Furthermore, a heatmap of selected type I IFN (IFN-I) related genes confirmed a robust IFN-I gene signature in severe COVID-19 PMNs, while LDGs from severe COVID-19 distinctively lacked this signature (Figure 1C). Unsupervised clustering analysis, namely Iterative Clustering and Guide Gene Selection (ICGS) using the AltAnalyze software, supported these findings by identifying the top 118 differentially expressed (DE) genes, including several IFN-related genes (Supplementary Fig. 1B). Similarly to the selected samples included in Figure 1, this analysis classified the samples into two major clusters: a first one containing all isolated LDG samples, and a second one comprising all isolated PMN samples. The former cluster consisted of neutrophil antimicrobial and granule marker genes (e.g. DEFA3, DEFA4, SERPINB10, CTSG), while in the latter cluster the most significantly upregulated genes in the PMNs from severe COVID-19 subgroup were mainly interferon inducible (e.g. IFI44L, IFI6, GBP3, IRF7). These differences were supported by a detailed gene analysis (Supplementary Fig. 2A).

Inflammasomes are activated in severe COVID-19 PMNs, but not directly by SARS-CoV-2

Looking more closely into PMN fractions, pathway analyses identified the inflammasome related NOD-like and RIG-like receptor signaling pathways among the most significantly overrepresented pathways, differentially expressed in severe COVID-19 PMNs versus HC PMNs (Figure 2A and Supplementary Fig. 2B-C) or mild COVID-19 PMNs (Figure 2B and Supplementary Fig. 2D, E). However, mild COVID-19 PMNs did not significantly differ from HC PMNs in their inflammatory profile (Supplementary Fig. 2F)."

Finally, to respond to the reviewer's specific question concerning whether the observed differences are still present in the absence of the newly defined low-purity samples, we can conclude that the results continue to highlight the differences we had previously described (increased cell cycle and metabolism-related pathways in LDGs and a distinct IFN-I signature in severe COVID-19 PMNs).

- While the in vitro data suggest that IFN-I may prime the inflammasome response in neutrophils, in vivo evidence remains inconclusive. The systemic blockade of IFNAR1 with antibodies in infected mice does not confirm that IFN-I directly primes neutrophil inflammasomes, as other cells could initially sense IFN-I and subsequently produce

neutrophil-activating stimuli. In the absence of in vivo experiments utilizing conditional IFNAR1 knockout models, such as Mrp8-Cre x IFNAR1 fl/fl mice, the authors should consider moderating the stated significance of these findings in the discussion about the limitations of the study.

Response*: We agree with the reviewer on this point. Thus, we modified the "limitations of study" paragraph in the discussion.*

Revised manuscript, discussion section, lines 674-677:

"Furthermore, the observed inhibitory effects on neutrophil inflammasome activity by IFNAR blockade does not exclude the possibility that IFN-I could promote neutrophil inflammasome formation by indirect effects such as stimulating the release of pro-inflammatory cytokines by other cell types."

- Given the reports of life-threatening COVID-19 infections occurring in conjunction with autoantibodies against type I IFNs (DOI: 10.1126/science.abd4585), the authors should explore how this intersects with their findings. A discussion is needed on whether patients with such autoantibodies may exhibit inflammasome activation patterns similar to the severe cases in this study, which could provide valuable insights into patient stratification and treatment approaches.

Response*: This is a good point, thank you. Based on our data it is unlikely that patients with IFN-I autoantibodies or genetic defects in the production of IFN-I would show significant neutrophil inflammasome activation. Like with IFN-I response in general, neutrophil inflammasomes can probably be either protective or damaging to the host, depending on the context and durability of the response. We have discussed this topic further in the revised manuscript, in response to this concern and the first point of reviewer 1.*

Revised manuscript, discussion section, lines 639-648:

"The dualistic nature of the IFN-I response in COVID-19 has been recognized previously. It seems that a strong initial IFN-I response to SARS-CoV-2 is more likely to result in asymptomatic or mild COVID-19 whereas a decreased initial IFN-I activity, due to e.g. genetic defects or increased levels of IFN-I autoantibodies, can lead to more severe COVID-19 (61). This initial beneficial effect of IFN-I is probably due to its ability to limit viral replication at early stages of the infection. However, at later stages of the disease IFN-I can be detrimental by promoting inflammatory pathways instead of direct antiviral effects (62). Thus, similarly to the IFN-I response in general, the role of neutrophil inflammasomes in development and severity of COVID-19 might be dualistic in nature with an initial protective effect while damaging when sustained for prolonged periods."

- The authors need to delve deeper into whether the inflammasome patterns observed in severe COVID-19 cases are a contributing factor to the disease's progression or a result of the infection's severity, thereby clarifying the causality in their discussion.

***Response**: This is an important aspect of the study and we agree that the relationship between neutrophil inflammasome activity and disease severity could be highlighted better. However, understanding causality by analyzing clinical patient samples is difficult due to the typical lack of patient samples from the early phase of the disease. Hospitalization and thereby patient sample collection typically occurs when the patients are already experiencing the peak of symptomatic phase of the disease. To highlight the link between neutrophil inflammasome formation and disease severity more clearly, we have marked the statistically significant correlations between inflammasome activation and disease severity parameters by an asterisk in the correlation plot (Fig. 5) and added text in the discussion accordingly.*

Revised manuscript, discussion section, lines 649-654:

"Our study demonstrated a strong association between PMN caspase1 activity and plasma levels of calprotectin, a marker of neutrophil activation. Additionally, increased disease severity, as assessed by the WHO ordinal scale, was significantly linked to PMNs being less responsive to ex vivo IFN-induced inflammasome activation, which is suggestive of prior in vivo inflammasome activation. Thus, these results suggest that neutrophil inflammasomes play a potential role in disease severity rather than being protective in COVID-19."

This point links to the previous comment by the reviewer regarding the role of IFN-I in disease severity. We have added text in the discussion highlighting the dual nature of the IFN-I response and neutrophil inflammasome activation in COVID-19 disease severity (see response above).

-Authors should clarify if and how their findings may lead to any therapeutic advantage for severe COVID-19 patients.

***Response:** The text has been adapted accordingly, and more details have been added into the revised manuscript.*

Revised manuscript, discussion section, lines 685-687:

"For example, pharmacologically targeting the inflammasome pathway in neutrophils with novel inhibiting molecules, may help mitigate the exaggerated inflammatory response observed in severe cases."

Reference added: Mangan, M. S., Olhava, E. J., Roush, W. R., Seidel, H. M., Glick, G. D., & Latz, E. (2018). Targeting the NLRP3 inflammasome in inflammatory diseases. Nature reviews Drug discovery, 17(8), 588-606.

Minor points:

-Authors should increase the font in all the figures as most of them are difficult to be read

Response: *Thank you for your suggestion. The font sizes have been increased, but their size will ultimately depend on the requirements of the journal where is the work will be published.* *Thank you for your suggestion. The font sizes have been increased, but their size will ultimately depend on the requirements of the journal where the work will be published.*

-Authors should better clarify how the choice for statistics tests was conducted "depending on sample distribution and the number of groups analyzed" in the Methods.

Response: *We added additional details to the methods.*

Revised manuscript, methods section, lines 329-331:

"To elaborate, nonparametric tests like Mann-Whitney and Kruskall-Wallis were employed when the data violated assumptions of normality, while ANOVA tests were applied when the data met parametric assumptions".

****Referee Cross-Commenting*****

-I agree with Reviewer#1 with adding relevant references about the evolution of some viruses, including SARS-COV-2, in evading type I IFN response.

Response*: We added this information into the manuscript.*

Revised manuscript, discussion section, lines 635-638:
"It should be noted that several SARS-CoV-2 encoded proteins have been shown to inhibit IFN-I signaling. However, no evidence suggests that neutrophils can be infected by SARS-CoV-2 and therefore it seems unlikely that such direct virus mediated effects could play a role in the observed neutrophil unresponsiveness to IFN-I".

Reference added: Rashid F, et al. Roles and functions of SARS-CoV-2 proteins in host immune evasion [preprint]. Front Immunol. 2022;13.

-I agree with Reviewer#1 about Figure 8 not delivering a novel message. I also suggest to move this to the supplementary section.

Response*: We have moved the figure plate to the Supplements.*

Revision Plan

4. Description of analyses that authors prefer not to carry out

Please include a point-by-point response explaining why some of the requested data or additional analyses might not be necessary or cannot be provided within the scope of a revision. This can be due to time or resource limitations or in case of disagreement about the necessity of such additional data given the scope of the study. Please leave empty if not applicable.
