# Supplementary material for: The assembly of neutrophil inflammasomes during COVID-19 is mediated by type I interferons
Source: PLoS Pathog. 2024 Aug 22;20(8):e1012368. doi: 10.1371/journal.ppat.1012368 (PMC11340896; doi:10.1371/journal.ppat.1012368)
Supplement: Attachment — Submitted filename: 080524-comments PPath.pdf [file ppat.1012368.s013.pdf]

Dear Dr. Cabrera, two additional expert reviewers have provided comments. They both view your report to be important and with potential to inform new therapies for COVID19. Patient samples and a focus on understudied cell types influencing COVD19 make this submission valuable to the field. However, the reviewers expected additional results that are necessary to identify the relative contribution of neutrophil inflammasomes to severe COVID19. First, both reviewers noted that flow cytometry can reveal multiple signatures of isolated PMNs, without requiring ex vivo stimulation. Such flow results can help explain how PMNs may operate in COVID19. Second, reviewer 2 recommended orthogonal approaches to measure caspase activation, as the results may address the spontaneous IL1beta releases and other discrepancies in the current data. Both of these experiments should be performed and the new results presented in a revised submission. Note that many of the minor issues may be suitably addressed with modifications to the text.

Reviewer #1: In their current revision, the authors made modifications according to previous review. The manuscript is improved in clarifying their major information. However, current data and discussion fail to offer the direct answer to the question raised by previous review: "what is the contribution of neutrophil inflammasomes to the severity of COVID-19". Although PMN isolated from severe cases show different characteristics in ex vivo experiments compared to those isolated from healthy donors, it is not hard evidence supporting their in vivo role. In addition, as pointed out by previous review, no clear effects of PMN inflammasome were found in animal model which further ruin the persuasion of authors' conclusion.

Response: *The reviewer is correct that "hard evidence" of neutrophil inflammasome activation in the animal model was lacking in the previous version of the manuscript. Therefore, for the revised manuscript, we have conducted additional mouse experiments, and detected caspase 1 activation in neutrophils by flow cytometry directly in lung single cell suspensions (without additional stimulus). Increased caspase 1 activity was observed at 4 dpi but not at 2 dpi (new Fig. 8A). We feel that these results corroborate our previous findings in which we detected increased caspase 1 activation after nigericin-mediated activation of isolated mouse neutrophils.*

Unfortunately, the authors declined the application of flow cytometry in their additional experiment. In addition to its quantitative function, flow cytometry can also show the expression of multiple molecular signatures of isolated PMN without further ex vivo stimulation, which may represent the real situation of PMN in severe COVID-19 cases and their healthy controls.

Response: *Admittedly, we were hesitant to performing flow cytometry solely for neutrophil quantification purposes, since other less cumbersome methods are available. However, the reviewer has now pointed out the lack of direct evidence for inflammasome formation in the mouse model (see comment above). Therefore, we have undertaken additional experiments and have employed flow cytometry to simultaneously quantify neutrophils and measure their caspase 1 activity. In addition, we used surface expression of CD11b as a maturation/activation marker for neutrophils. By these means, we confirmed the previously observed neutrophil accumulation in the lungs (new Fig. 7D-F) and observed increased caspase 1 activity directly in neutrophils of infected mice (as discussed above). We also noted a decrease in CD11b surface expression in neutrophils of infected mice, which we think is more likely due to activation of mature rather than increased*

accumulation of immature neutrophils (nuclear staining of isolated neutrophils displayed primarily multilobed nuclei indicative of mature neutrophils, Fig. 7G-H).

Finally, the authors refined their discussion on the different roles of PMN and LDG. However, the LDG data is still ambiguous and doesn't help clarify the role of PMN inflammasome in severe COVID-19. I would recommend removing them at all.

Response: *We agree that our LDG data does not directly clarify or explain the role of PMN inflammasomes during COVID-19. In addition, due to the lower frequency of LDGs as compared to PMNs in blood during COVID-19, it is unlikely that LDG inflammasomes would have a strong impact on COVID-19 pathogenesis. However, we feel that our LDG data address important questions concerning the life cycle of neutrophils by indicating dynamic changes in the outcome of inflammasome activation between LDGs and PMNs that are most likely related to maturation (e.g. the observed significant defect of PMNs to release IL-18 upon inflammasome activation). Therefore, we feel that the LDG data is of interest from the neutrophil biology perspective, while not necessarily better explaining the COVID-19 pathogenesis directly.*

Ultimately, the purpose of the LDG data is not to clarify the role of PMNs but to study inflammasome activation in neutrophils and include distinct populations that are currently less well described in the literature. Although a characterization of LDG inflammasomes as thorough as PMN inflammasome was not possible due to the scarcity of the cell population, we believe the insight provided remains relevant. Moreover, we agree with reviewer #2 when stating that: "the specific interrogation of inflammasome pathways in low-density granulocyte (LDGs) versus mature neutrophils (PMNs) and mechanistic involvement of IFN signaling in this pathway is novel". Although many studies have described an increase in circulating immature neutrophils during COVID-19 and other viral infections, illustrating their different inflammatory profile (from mature PMNs) has not been the focus of other studies.

Reviewer #2: Overall, this is an exciting paper that provides elegant RNA sequencing data from patient samples with ex vivo assays and an in vivo mouse infection model that further characterize potential mechanisms of inflammasome activation in neutrophils in response to SARS-CoV-2 infection/COVID-19. Although NLRP3 inflammasome activation has been demonstrated in neutrophils previously, the specific interrogation of inflammasome pathways in low-density granulocyte (LDGs) versus mature neutrophils (PMNs) and mechanistic involvement of IFN signaling in this pathway is novel. Importantly, the scope of this work extends beyond COVID-19, as similar pathways are likely to be activated in neutrophils in other viral infection models or inflammatory diseases.

The paper would benefit from additional assays to confirm caspase-1 activation (such as ASC-speck formation, FLICA, or WB for caspase-1 or Gasdermin-D cleavage) in mouse neutrophils isolated from infected lungs and in COVID-19 PMNs, which may help resolve discrepancy between the spontaneous IL-1b release measured (fig. 2C) despite no detectable caspase-1 activation (as measured by luciferase) in the absence of stimulation (fig. 2G). Other experiments suggested, including more robust phenotypic characterization of the neutrophils recruited to the mouse lungs in the in vivo Sars-CoV-2 infection model by flow cytometry would also improve the manuscript.

Response: *We would like to thank the reviewer for these constructive comments. To address the points raised, we conducted additional mouse experiments and performed flow cytometry on mouse lung single cell suspensions to quantify neutrophils and measure their CD11b surface expression levels as a marker of neutrophil maturation/activation. Furthermore, we employed flow cytometry to detect caspase 1 activity directly in lung neutrophils (without exogenous stimulus) by using a FAM-FLICA fluorescent probe. The results confirm the previously observed neutrophil accumulation in the infected lungs (new Fig. 7D-F). We also observed a decrease in CD11b surface expression in neutrophils of infected mice, which was found to be more likely due to activation than increased accumulation of immature neutrophils (nuclear staining of isolated neutrophils from infected mice displayed primarily multilobed nuclei indicative of mature neutrophils (Fig. 7G-H). Importantly, we also observed increased caspase 1 activity in neutrophils in infected lungs, at 4 dpi (but not at 2 dpi, new Fig. 8A). We feel that these results corroborate our previous findings, in which we detected increased caspase 1 activation after nigericin-mediated activation of isolated mouse neutrophils.*

Regarding the points raised concerning human PMNs, please see response below.

Major comments:

Reviewer #2: Fig 2C shows spontaneous IL-1b release (i.e. no signal 2 required) in COVID-19 PMNs but no difference in caspase-1 activation in Fig 2G - only see casp-1 activation in response to nigericin. But IL-1b release is presumably caspase-1 dependent. Can the authors measure caspase-1 activation by another approach? Such as staining for ASC-specks, FLICA, or WB (looking at caspase-1 or gasdermin D cleavage)? Any additional assay confirming caspase-1 activity in COVID-19 PMNs would increase rigor and add significantly to the paper.

Response: *This is a good point and we agree that IL-1b release is presumably caspase 1 dependent. In fact, when looking more closely at non-treated mock infected mice in Fig 2G, there is a trend of increased caspase 1 activity in COVID-19 PMNs as compared to HC, which then becomes statistically significant after treatment with nigericin. This suggests that while observable caspase-1 activity might not differ in baseline at least when using the luminescence method as the read-out, the response to specific stimuli like nigericin is heightened in COVID-19 PMNs.*

For this reason, as the reviewer points out, another more sensitive method to detect caspase 1 activity would be beneficial. Unfortunately, due to the season-dependent scarcity of new COVID-19 cases in Finland, we currently cannot repeat these assays with fresh human samples.

However, we performed additional experiments on HC PMNs to show that IFN-I can directly cause caspase 1 activation and IL-1b release, which could at least to some extent increase our understanding of the previous results which showed spontaneous IL-1b release with concomitantly low or undetectable levels of caspase 1 activity in COVID-19 PMNs. These new assays showed increased release of IL-1β after treating HC PMNs with IFN-I or LPS (without the 2nd signal) for an extended period of 24 h (similar to what was used for COVID-19 PMNs) (new Fig. 4M). Importantly, by using the FAM-FLICA fluorescent probe, we could detect increased caspase 1 activity by treating HC PMNs with a high concentration of IFN-I or LPS (but not the "normal" amount of IFN-I used in our other assays) after a short 4 h incubation period (new Fig. 4K). These results suggest that IFN-I can activate caspase 1 and IL-1β in a concentration-dependent manner

and as shown for the normal IFN concentration, IL-1β release can be detectable despite the absence of significant caspase 1 activity in our experimental setup.

Fig 4B seems to conflict with Fig 2C and Fig 2G, which shows COVID-19 PMNs display spontaneous IL-1b release and casp-1 activation in response to nigericin, respectively. In Fig 4B, COVID-19 PMNs (mock or nigericin only treated) don't exhibit any increased IL-1b release. This discrepancy should be discussed.

Response: *Yes, the reviewer is right. Indeed, there is a difference in spontaneous release of IL-1b between Fig. 2C and Fig. 4B. However, this difference is explained by the different incubation times in the experiments. For Fig. 2C, the incubation time was 24 h while for Fig. 4B it was only 8 h. We have tried to make this clearer in the revised manuscript by replacing "overnight" with "24 hours" on lines 139 282, 437 and 524.*

The authors state that "neutrophils may lose the ability to secrete IL-18 during maturation" (lines 511-512). Can the authors directly compare cytokine production between HC PMNs, COVID-19 PMNs and COVID-19 LDGs (+/- stimulation)? This data would better support the conclusion drawn, as the following discrepancies contradict this conclusion: In Fig 2, there is no difference in IL-18 secretion between HC and COVID-19 PMNs, but the raw values are ~50pg/mL. In Supplement 4D, there is no difference between stimulated and unstimulated HC PMNs, raw values ~40 pg/mL. In Fig 6, the raw values of IL-18 release from LPS+nig-stimulated COVID-19 LDGs is also ~50 pg/mL, but appear higher relative to untreated LDGs, which exhibit little-to-no cytokine production. Taken together, it doesn't seem like neutrophils are losing their ability to secrete IL-18 as they differentiate. Plos does not accept references to data not shown (line 511), but if the authors show these data, it would be a good demonstration to support the above conclusion that inflammasome-dependent cytokine release in neutrophils is dependent on their differentiation status

Response: *Yes, it is true that PMNs express IL-18 constitutively. We have recognized this in the current manuscript in two occasions, in lines 500-501: "Furthermore, the observed residual IL-18 was not affected by inflammasome inhibitors, suggesting its secretion to be unrelated to inflammasome activity in PMNs"; and "To note, PMN's lack of IL-18 secretion is not due to lack of protein, as they constitutively express significant amounts intracellularly (63)". For the revised manuscript, we modified the sentence in lines 578-579 to "Overall, these findings suggest that neutrophils may lose the ability to secrete IL-18 in response to inflammasome activity during maturation".*

The in vivo experiments using the mouse Sars-CoV-2 infection model are critical to validate the author's RNA seq analyses and ex vivo characterization of neutrophil populations in COVID-19, and therefore, Fig 7 would benefit from additional data. Can the authors perform flow cytometry on cells in the lung to 1. validate neutrophil recruitment during infection (quantification of Ly6G-pos cells), 2. phenotype these cells (are the Ly6G-pos cells mature or immature neutrophils?) and 3. quantify caspase-1 activation in lung neutrophils during infection (using a caspase-1 Flica-probe or by measuring ASC specks in isolated neutrophils). As an alternative approach, are inflammasome-dependent cytokines upregulated in the BALF 2 dpi (when peak neutrophil recruitment is measured) and is cytokine production inhibited by the anti-IFNAR treatment?

Response: *We thank the reviewer for these very constructive suggestions. We have performed additional experiments accordingly (as already discussed in the above responses). Most new data is*

included in the new Fig.7, which describes neutrophil accumulation in the infected mice or incorporated in the new Fig. 8 (previously Fig. 7), which describes IFN-dependent caspase 1 activity in mice. We decided to use the FAM-FLICA probe instead of an ASC-speck based analysis due to its compatibility with flow cytometry. Also, as the reviewer pointed out below, ASC-specks are not always involved in inflammasome activation and/or can be difficult to detect in neutrophils. We considered also the other suggested approach of measuring inflammasome-related cytokines in infected mice. However, in this approach it might be difficult to discriminate neutrophil from macrophage inflammasome activity and thus we decided to use flow cytometry instead.

Minor comments:

Reviewer #2: In lines 409 – 410, the authors state that the non-COVID-19 patients presented with ARDS and clinical concern for COVID-19 but tested negative for SARS-CoV2 by PCR. It is not clear if the 'non-COVID-19 patients were once seropositive, and this distinction is important for the conclusion drawn. Perhaps more accurate terminology would be Sars-CoV-2 PCR positive vs. PCR negative?

Response: *We think we should use this same terminology as they use in the cited LaSalle et al. paper. However, we amended our text to make it clear that the distinction between COVID-19 and non-COVID-19 is based on SARS-CoV-2 PCR: "The non-COVID-19 patients exhibited acute respiratory distress and clinical suspicion for COVID-19. However, they tested negative for SARS-CoV-2 by PCR, unlike those classified as COVID-19 patients" (Revised MS, lines 458-460).*

Could the authors provide a list of genes for the pathways shown in Figure 3, provided as a supplement?

Response: *Thank you for your suggestion. The gene set enrichment analysis shown in Figure 3 made use of the pathway sets publicly available in the Molecular Signatures Database (or MSigDB). A detailed list of genes was added to the revised manuscript as Supplementary Table 1, as well as a sentence in the materials and methods section.*

Revised manuscript (Materials and methods section, lines 210-211):

"For the GSEA of the reanalyzed RNA-seq data from LaSalle et al. (8), we used the fgsea R package using MSigDB pathway sets, as specified in Supplementary Table S1."

The authors conclude that Sars-CoV-2 particles have no (direct) effect on PMN inflammasome activation, but viral particles are only used as 'signal 2' in these experiments and IL-1b release appears to be nigericin dependent. Did the authors test if viral ligands instead prime PMNs for inflammasome activation? Is IL-1b released from HC PMNs stimulated with Sars-CoV-2 particles + nigericin?

Response: *We have used the virus also as the first signal in our preliminary experiments. However, we did not find evidence that the virus can act as the first signal for inflammasome activation by nigericin (representative figure shown below). Thus, evidence showing both that IFN-I is able to act as the first signal in vitro and that patient PMNs showed strong IFN-I signature, led us to conclude that IFN-I is the more likely mode of neutrophil inflammasome priming during COVID-19.*

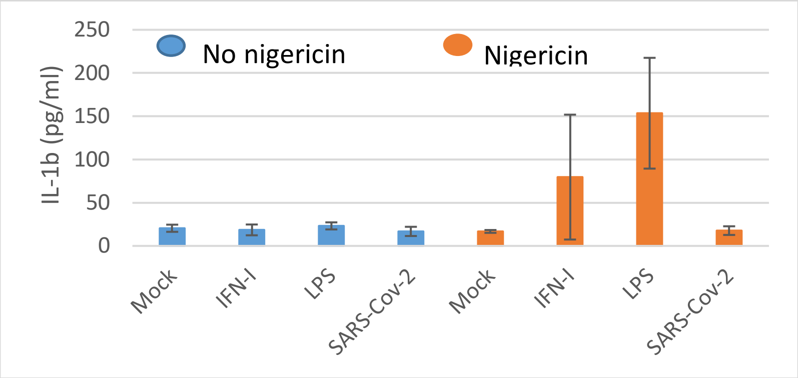

Could the authors further investigate the relationship between calprotectin and inflammasome activation in Fig. 5?

1. Is S100A8 and A9 upregulated in the RNAseq data set in severe COVID-19 PMNs?

Response: *Yes. This is visible in the volcano plots in Supplementary Figure 2B, where both genes are upregulated in COVID-19 PMNs versus HC PMNs; and in Supplementary Figure 2E, where S100A8 is upregulated in severe versus mild COVID-19 PMNs.*

2. Since calprotectin levels are elevated in COVID-19 patients, it would be interesting to see if calprotectin can directly activate the inflammasome in neutrophils. Can the authors treat PMNs with S100A8/A9 (+/-nigericin) and measure caspase-1 activation or cytokine production?

Response: *The studies by Sreejit et al. in 2020 and 2021 have demonstrated the role of S100A8/A9 in activating the NLRP3 inflammasome in neutrophils, which is relevant to the reviewer's question regarding calprotectin (S100A8/A9) activating the inflammasome in neutrophils in the context of COVID-19. The 2020 study shows that S100A8/A9, released by neutrophils at the site of myocardial infarction, binds to Toll-like receptor 4 and primes the NLRP3 inflammasome in naïve neutrophils, thus promoting IL-1β secretion. This process stimulates granulopoiesis by acting on hematopoietic stem and progenitor cells in the bone marrow, and genetic or pharmacological disruption of S100A8/A9 signaling was shown to suppress it. In the 2021 study, it is further elucidated that the retention of NLRP3 inflammasome-primed neutrophils in the bone marrow is essential for the inflammatory response, highlighting the complex role of neutrophils and the NLRP3 inflammasome in inflammatory diseases. Together, these findings suggest that S100A8/A9 can indeed activate the NLRP3 inflammasome in neutrophils, leading to caspase-1 activation and cytokine production, which may have implications for COVID-19 patients with elevated calprotectin levels. The specific mechanism involves S100A8/A9 priming the NLRP3 inflammasome and the subsequent release of IL-1β, a process that can be enhanced by additional stimuli providing the second signal for inflammasome activation.*

We added the following to the discussion in the revised MS (lines 739-742): "". It is of interest to note that calprotectin can also promote inflammasome activity in neutrophils (Sreejit et al. 2020; Sreejit et al. 2021). Therefore, in addition to IFN-I discussed in this study, it is possible that calprotectin also contributes to neutrophil inflammasome formation during COVID-19".

3. Alternatively, does inflammasome activation promote S100A8/A9 release from neutrophils? This was recently demonstrated in PMID 37903858. It would be interesting to see if ex vivo LPS/IFN + nig stimulation of HC PMNs drives S100 production.

Response: *We agree that it could be interesting to study whether calprotectin could be released together with IL-1β upon inflammasome activation. In fact, based on the mentioned study, it seems that soluble calprotectin levels would likely be increased by stimulating neutrophils with LPS+Nig, which is known to activate the canonical NLRP3 inflammasome. However, we feel that this is a bit distant from the context for our current study and should rather be performed as part of follow-up experiments in which the molecular mechanisms and pathways of IFN-I dependent inflammasome formation are studied in more detail.*

Fig 5C legend should be IFN + nig (not LPS)

Response: *Thank you for spotting this mistake. It has been corrected in the revised MS.*

Supplementary Fig 5 (lines 492-493) – ASC transcript expression does not confirm inflammasome forming capacity of cells; inflammasomes can form in the absence of ASC

Response: *This is good point, thank you. We have revised our conclusions accordingly: Briefly, PYCARD gene coding for the ASC protein was expressed similarly in mature and immature neutrophils (Supplementary Fig. 5), suggesting that both cell types may have ASC-dependent inflammasome forming capacity.*

Line 502 - Wrong fig reference? Should be D-E

Response: *This has been corrected.*

In Supplement 6A and 7B, can the authors quantify viral load by PFU or genome copy number by qRT-PCR?

Response: *We titrated infectious virus in mouse lungs by measuring fluorescent focus forming units (FFU) in Vero E6 cells. These data are now presented in Fig. 7B and supplementary Fig. 7C.*

In Supplement 6B, viral antigen quantification should also be shown for PBS (as done in panel C).

Response: *It is true that IHC staining can result in false positive signal and this data should be shown. As mentioned above, we repeated the animal experiments and included this data in the new Fig. 7C.*

The authors conclude that their findings "suggest a pivotal role of neutrophils in clearing the virus in SARS-CoV-2 infected mice" (lines 534-535), when viral clearance was not measured. The data demonstrate neutrophil recruitment to the lungs in this mouse model, not a pivotal role for neutrophils in clearing viral infection.

Response: *We agree with the reviewer that this statement was an overinterpretation of our data and have removed it.*

Supplemental data depicting purity of isolated neutrophils (by flow cytometry) for Fig 7 should be included.

Response: *We added a representative flow cytometry histogram showing the number of LY6G+ neutrophils in the isolated cell fraction (supplementary Fig. 6C)*

In Supplement 7C, representative H3cit IHC staining should be shown for PBS/mock-infected mice; quantifications should also be shown, as in Supplement 6

Response: *- We have not included any image of a mock infected control mouse stained for H3cit, as there is no reaction at all. In control lungs, neutrophils (Ly6G+) are only seen as individual cells in the lumen of vessels, and there was no H3cit (NET) expression. This information is provided in Supplemental Table 3C where the findings in individual animals in Exp 3 are described. In our opinion, staining for H3cit rather represents proof of degeneration and NETosis of neutrophils occurring in infected animals; however, as its extent varied substantially between lung sections in infected and treated groups, we did not pursue a morphometric analysis.*
