# Supplementary material for: The assembly of neutrophil inflammasomes during COVID-19 is mediated by type I interferons
Source: PLoS Pathog. 2024 Aug 22;20(8):e1012368. doi: 10.1371/journal.ppat.1012368 (PMC11340896; doi:10.1371/journal.ppat.1012368)
Supplement: Attachment — Submitted filename: comments PPATH v3-100624.pdf [file ppat.1012368.s014.pdf]

Reviewer Comments (if any, and for reference):

Reviewer's Responses to Questions

Part I - Summary

Please use this section to discuss strengths/weaknesses of study, novelty/significance, general execution and scholarship.

Reviewer #1: Cabrera et al's manuscript "The assembly of neutrophil inflammasomes during COVID-1 19 is mediated by type I interferons" demonstrates the inflammatory characteristics of neutrophils in COVID-19 patients and SARS-CoV-2-infected mice. The study is interesting and consists of an impressive amount of data. However, the paper needs to be further organized to highlight the major findings. Currently, the authors describe the changes of PMN, LDG, inflammasome, and IFN-I by using both human COVID-19 patient samples and mice model, but it is hard to identify the main theme of the story.

Response: **Thank you for addressing these important concerns. We agree that the organization of our manuscript could be improved. Therefore, we made efforts in the newly revised manuscript to highlight the focus of the story as detailed in the answers to the point-to-point responses below. Hopefully these changes could help readers to identify the main themes of our manuscript.**

Reviewer #2: All comments were adequately addressed. New experiments added demonstrating caspase-1 activation in lung neutrophils isolated from infected mice add significantly to the paper.

Part II – Major Issues: Key Experiments Required for Acceptance

Please use this section to detail the key new experiments or modifications of existing experiments that should be absolutely required to validate study conclusions.

Generally, there should be no more than 3 such required experiments or major modifications for a "Major Revision" recommendation. If more than 3 experiments are necessary to validate the study conclusions, then you are encouraged to recommend "Reject".

Reviewer #1:

1. I agree with Reviewer 1 that "no clear effects of PMN inflammasome were found in animal model". In vivo depletion or blockade assay is needed.

Response: It is true that we have not addressed the contribution of PMN inflammasomes in the severity of the animal disease model and admittedly the assays suggested by the reviewer might help in this regard. In fact, our intention in the current manuscript is not to directly claim a causative effect but rather only indicate an association between PMNs inflammasomes and COVID-19. We apologize for not being clear enough with this message and we have tried to modify the text to make this point clearer (including the abstract). In addition, we have amended the limitations of our study to the discussion.

Revised manuscript, abstract section, lines 42-43:

"These findings suggest a potential role for neutrophil inflammasomes in driving inflammation during severe COVID-19."

Revised manuscript, discussion section, lines 779-784:

"Importantly, our results do not directly assess the role of neutrophil inflammasomes in COVID-19 pathogenesis in humans or in the animal disease model. Further studies are therefore needed to understand the relative contribution of neutrophil inflammasomes in COVID-19 disease progression as compared to the better described macrophage inflammasomes as well as to other inflammatory pathways engaged by neutrophils such as degranulation, reactive oxygen species production and NETosis".

2. I also agree with Reviewer 1 on "the authors refined their discussion on the different roles of PMN and LDG. However, the LDG data is still ambiguous and doesn't help clarify the role of PMN inflammasome in severe COVID-19". If removal of these data does not apply, the better organization is necessary.

Response: We agree that the LDG data does not explain COVID-19 pathogenesis. However, we feel that by providing a reference to PMNs the LDG data does play a role in emphasizing a unique pro-inflammatory role for mature neutrophils during COVID-19 (e.g. the lack of IL-18 expression in PMNs). We do agree with the reviewer that better organization could help make our message clearer and we therefore decided to change the structure of the newly revised manuscript in a way that would highlight the inherent differences between LDGs and PMNs. The results section of the revised manuscript begins by describing all RNA-seq data in Figures 1-3, followed by in vitro data and animal experiments. We have also added a few sentences in the beginning of several paragraphs of the results section to explain the rationale of the performed experiments more clearly while also discussing the LDG data in the discussion.

Revised manuscript, discussion section, lines 693-697:

"Distinctively from PMNs, LDGs do not display the IFN-I signature or upregulation of major inflammasome related genes, which indicates their lower responsiveness to IFN-I during COVID-19. However, since we were able to show that LDGs can form inflammasomes when stimulated by IFN-I ex vivo, their lower responsiveness is probably due to LDGs not being similarly exposed to IFN-I as compared to PMNs during COVID-19."

The changed figure order in the revised manuscript is as follows:

Fig. 1 remains as previously: Deals with the overall differences between PMNs and LDGs (including the IFN-I signature severe COVID-19 PMNs).
New Fig. 2: A combination of parts of previous Figs 2 and 6: Describes the differential inflammasome related gene expression between LDG and PMNs.
Fig. 3 remains as previously: Highlights the difference between COVID-19 and HC PMNs based on RNAseq published by La Salle et al.
New Fig. 4, previously part of Fig. 2: *Ex vivo* data describing inflammasome activation in freshly isolated COVID-19 PMNs.
New Fig. 5, previous Fig. 4: Ex vivo data describing IFN-I as priming factor for PMNs.
New Fig. 6, previous Fig. 5: Association between PMN inflammasome activation (as analyzed in Figs 4 and 5) and disease severity.

New Fig. 7, part of previous Fig. 6: Ex vivo data highlighting the differential inflammasome activation induced cytokine expression in LDGs as compared to PMNs.
New Fig. 8, previous Fig. 7: Neutrophil accumulation in SARS-CoV-2 infected mouse lungs.
New Fig. 9, previous Fig. 8: Caspase1 activation in neutrophils of SARS-Cov-2 infected mice.

3. In addition, if the authors' explanation (increased IL-18 production in LDG results from its constituent expression) is correct, then type I IFN is dispensable for the universal inflammasome expression of neutrophils (including PMN and LDG), which impair the conclusion of this manuscript.

Response: We have failed to locate the referred text "increased IL-18 production in LDG results from its constituent expression" anywhere in the manuscript text or in responses to reviewer's comments. What we do mention in lines 767-768 is that "PMN's lack of IL-18 secretion is not due to lack of protein, as they constitutively express significant amounts intracellularly", referring to the PMNs and not LDGs. However, in case we have missed this, it would be a mistake and it should read "increased IL-18 production in LDGs compared to PMNs upon ex vivo stimulation is probably due to their higher expression of IL-18 mRNA".

4. In addition to flow cytometry, identifying the phenotype of neutrophils in lung slides by confocal microscopy may help illustrate their potentially pathogenic roles during SARS-CoV-2 infection.

Response: We agree that further experiments would be interesting and could provide more information on the exact role neutrophils play during the disease. However, we feel these additional assays could be performed as follow-up experiments, in which we (or someone else) would dissect the role of neutrophil inflammasomes in COVID-19 pathogenesis in much greater detail.

5. Finally, the blockade of IFNAR is not cell-specific. Therefore, the blocking effects on other immune cells, either quantity or quality, need to be addressed.

Response: True, IFNAR blockade is not cell-specific as IFN-I affects almost all cell types. Therefore, while we observed diminished neutrophil inflammasome activation by blocking IFNAR in SARS-CoV-2 infected mice, we cannot exclude any indirect mechanisms by which IFN-I could regulate neutrophil inflammasomes. We have therefore amended the limitations of our study section in the discussion.

Revised manuscript, discussion section, lines 784-791:

"Furthermore, due to the ubiquitous expression of IFNAR, the observed inhibitory effects on neutrophil inflammasome activity by IFNAR blockade does not exclude the possibility that IFN-I could promote neutrophil inflammasome formation by indirect effects such as regulating the interplay between neutrophils and other immune cells or stimulating the release of pro-inflammatory cytokines by other cell types. In addition, investigating the effects of IFNAR blockade in other time points than the chosen 2 dpi might have been more valuable in revealing its effects on viral replication and neutrophil accumulation in the infected lungs."

NOTE: Changes in the text of the manuscript have been highlighted in yellow, while whole paragraphs moved from their previous location (along with the figure changes) are marked in a green underlined text.
